# Tailored oxido-vanadium(V) cage complexes for selective sulfoxidation in confined spaces†
†Electronic supplementary information (ESI) available: Materials and instrumentations, synthesis and characterization, experimental details, catalytic kinetic data, ^1^H NMR titrations, *etc.* See DOI: 10.1039/c6sc03045a


**DOI:** 10.1039/c6sc03045a

**Published:** 2016-09-05

**Authors:** Dawei Zhang, Kelsey Jamieson, Laure Guy, Guohua Gao, Jean-Pierre Dutasta, Alexandre Martinez

**Affiliations:** a Shanghai Key Laboratory of Green Chemistry and Chemical Processes , School of Chemistry and Molecular Engineering , East China Normal University , 3663 North Zhongshan Road , Shanghai , 200062 , P. R. China; b Laboratoire de Chimie , École Normale Supérieure de Lyon , CNRS , UCBL , 46 allée d’Italie , F-69364 Lyon , France; c Aix Marseille Univ , CNRS , Centrale Marseille , iSm2 , Marseille , France . Email: alexandre.martinez@centrale-marseille.fr

## Abstract

Five sets of oxido-vanadium(V) complexes, which include both cages and open structures, were prepared and tested in the catalytic oxidation of sulfides. It was found that the hemicryptophane complexes, which are simultaneously comprised of cyclotriveratrylene (CTV), binaphthol and oxido-vanadium(V) moieties, are the most efficient supramolecular catalysts. The specific shape of the confined hydrophobic space above the metal center leads to a strong improvement in the yield, selectivity and rate of the reaction, compared to the other catalysts investigated herein. A remarkable turnover number (TON) of 10 000 was obtained, which can be attributed to both the high reactivity and stability of the catalyst. Similarly to enzymes, the kinetic analysis shows that the mechanism of oxidation with the supramolecular catalysts obeys the Michaelis–Menten model, in which initial rate saturation occurs upon an increase in substrate concentration. This enzyme-like behavior is also supported by the competitive inhibition and substrate size-selectivity observed, which underline the crucial role played by the cavity.

## Abstract 2

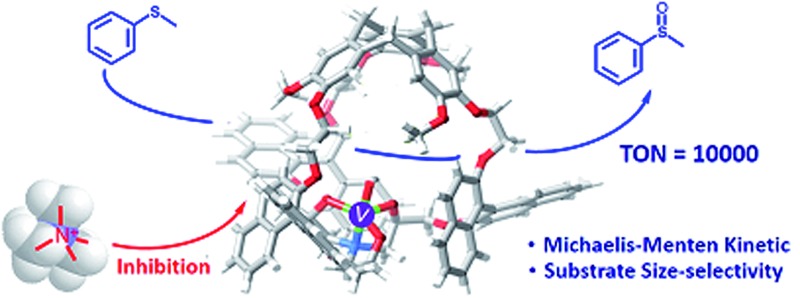
An oxido-vanadium(V) site encapsulated in a highly confined space of molecular cage displays enzyme-like catalytic behaviour.

## 1. Introduction

Molecular cages, which bear a well-defined cavity with suitable endohedral functionalities, are very attractive since they can mimic biological systems, such as enzymes, to realize supramolecular catalysis of various reactions in their confined space.^1^ These cage catalysts are capable of surrounding the surfaces of reactants and impose a specific orientation and conformation to the substrate in the vicinity of the catalytic site, thus leading to new and original reactivity.^2^ Importantly, the goal of using such systems as true synthetic analogues of enzymes requires not only high selectivity but also enhancement of reaction rates, like in enzymatic reactions.^3^ However, product inhibition using supramolecular catalysts is commonly observed, since the high affinity of the product with the cavity can prevent further catalytic cycles.^4^ Consequently, relatively large catalyst loadings are frequently required and low turnover numbers (TONs) are usually obtained. This issue has become one of the biggest challenges in supramolecular catalysis.^4*a*^ A pioneering work presenting high-turnover catalysis with supramolecular catalysts was reported by the Raymond and Bergman groups in 2011.^5^ They incorporated a ruthenium(ii) cation into a supramolecular assembly, which was active in the isomerization of allyl alcohols with turnovers reaching 1070. Recently, using the same assembly, high TONs (>300) were also reached in alkyl–alkyl reductive elimination catalyzed by encapsulated gold(iii) or platinum(iv) complexes.^2*j*^

**Fig. 1:**
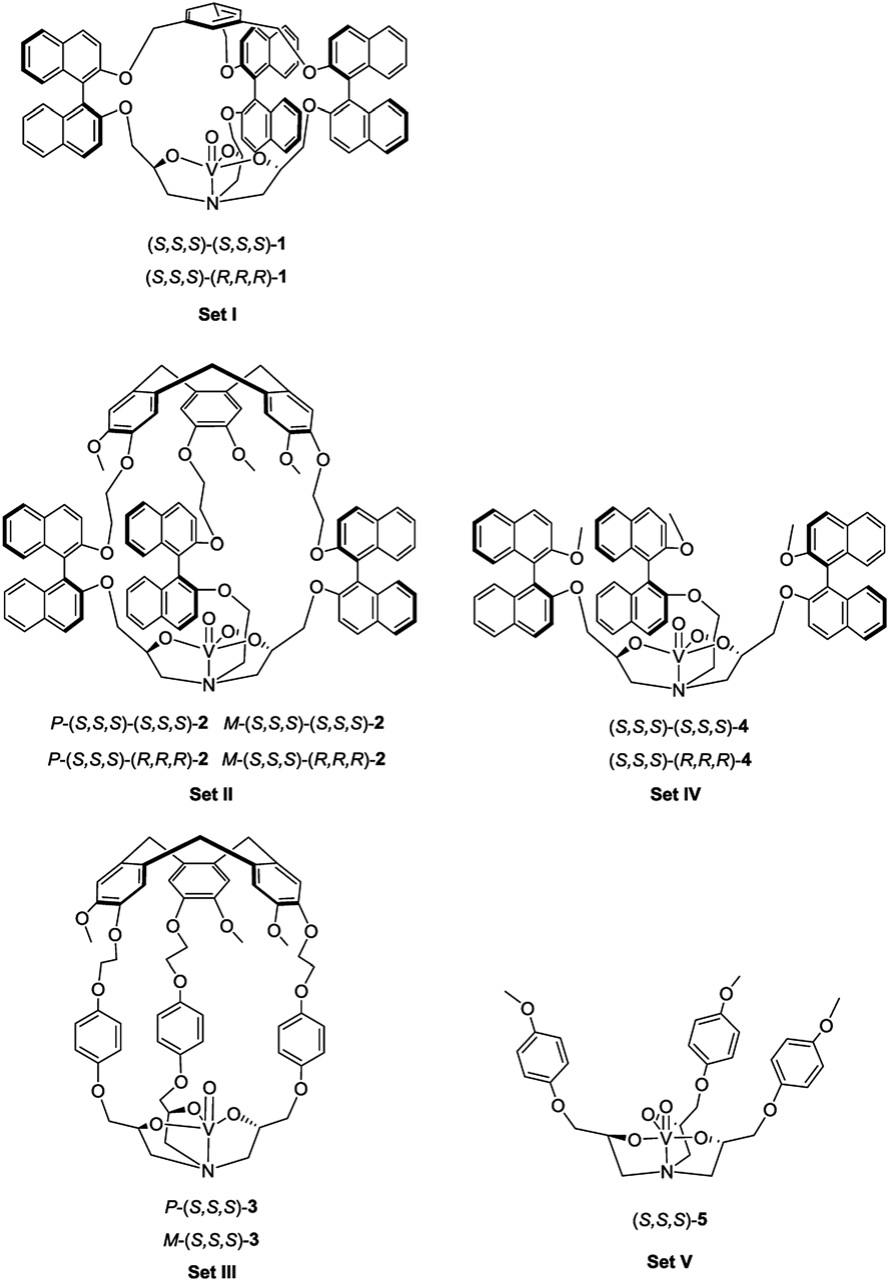
Structures of the five sets of oxido-vanadium(V) complexes.

Although tremendous advances have been made in the development of self-assembled hosts for guest encapsulation and transformation, it should be noted that covalent cages present several advantages, such as their stability and easy endohedral functionalization.^2n–p,6^ Among the covalent cage compounds hemicryptophanes, which are constructed from a cyclotriveratrylene (CTV) moiety and one other different *C*_3_ symmetrical unit, have recently received growing interest. The endohedral functionalization of their cavities *via* covalent bonds is versatile and gives rise to various excellent supramolecular catalysts which present improved selectivity, reactivity, TON and/or stability compared to the related model catalysts which lack a cavity.^7^ For instance, an oxido-vanadium(V) active site was attached inside the hydrophobic cavity of hemicryptophanes (complexes in Set III, Fig. 1), which were efficient in the oxidation of a hydrophobic sulfide substrate into the hydrophilic sulfoxide product with 180 turnovers.^7*e*^

In the present work, we have designed hemicryptophane derivatives containing binaphthol moieties (complexes in Set II, Fig. 1), with the aim of constructing more hydrophobic cavities. In addition, compared with the catalysts in Set III, the location of the bulky binaphthol units in the linkers can isolate more efficiently the heart of the cavity from the bulk solution, thus the effect of the confinement in stabilizing a transition state should be more pronounced. We have also prepared the oxido-vanadium(V) complexes of the related open structure (complexes in Set IV and V) and of cages with a smaller cavity size (cage complexes in Set I) to demonstrate the necessity of having a cavity and the essential role of its size. Sulfoxidation, which is an important oxidation reaction due to the importance of sulfoxides in the synthesis of pharmaceutical and agrochemical products,^8^ was chosen as the model reaction. It was found that the new hemicryptophane complexes (Set II) combining both a CTV moiety and binaphthol units are most efficient for the oxidation of thioanisol, and exhibit a TON of 10 000, which is a remarkable TON for a supramolecular cage catalyst. Mechanism studies show that the catalytic behaviors exhibit Michaelis–Menten kinetics, competitive inhibition and substrate size-selectivity, which suggest that the reaction occurs inside the cavity of the hemicryptophanes.

## 2. Results and discussion

### 2.1 Design and synthesis

**Scheme 1:**
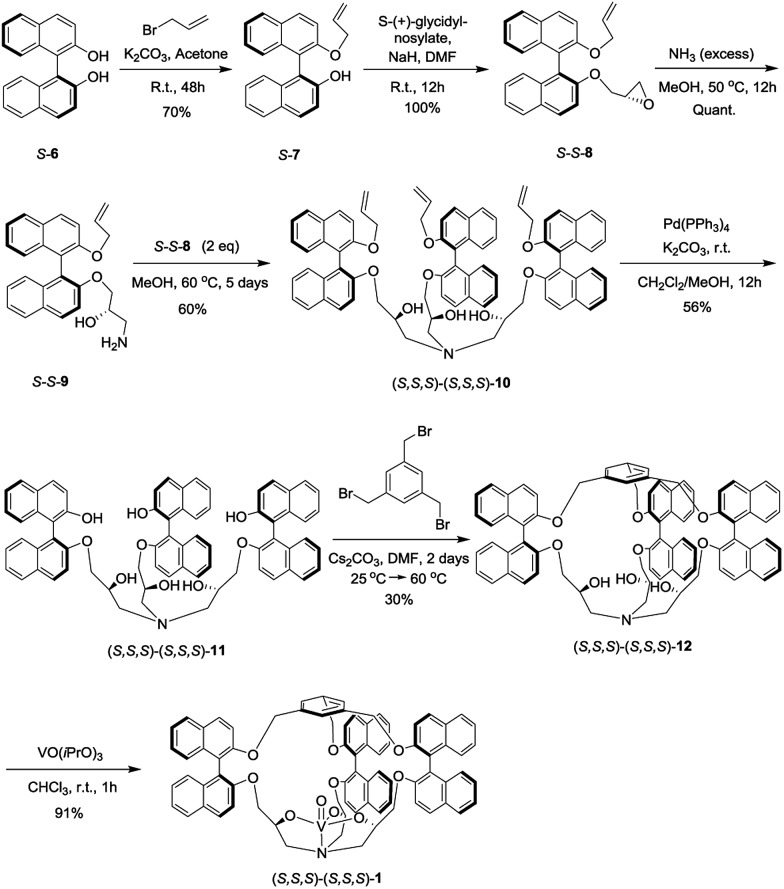
Synthesis of the complex (*S*,*S*,*S*)-(*S*,*S*,*S*)-**1**.

The synthesis of the oxido-vanadium complexes **3** and **5** has been previously reported.^7e,9^ Complexes **2** and **4** were obtained from the corresponding stereoisomers^10^ by reaction with vanadium(V)-oxytriisopropoxide in CHCl_3_ at room temperature (r.t.) with excellent yields (>90%). The ^1^H NMR spectra of each isomer of complexes **2** and **4** are consistent with a *C*_3_ symmetry. The synthetic route for the vanadium complexes in set I, such as (*S*,*S*,*S*)-(*S*,*S*,*S*)-**1**, is shown in Scheme 1. Firstly, the enantiopure binaphthol 6 was mono-protected with an allyl group *via* the reaction of *S*-**6** with 1 equiv. of allyl bromide in acetone in the presence of K_2_CO_3_ to give *S*-**7**. The enantiopure epoxide (*S*,*S*)-**8** was obtained by the regioselective nucleophilic substitution reaction of *S*-**7** on commercially available (*S*)-(+)-glycidyl nosylate in DMF. An excess of ammonia in methanol was then reacted with (*S*,*S*)-**8** to give the primary amine (*S*,*S*)-**9**. The *C*_3_ symmetrical allyl-protected trialkanolamine (*S*,*S*,*S*)-(*S*,*S*,*S*)-**10** was obtained by the trimerization reaction of **9** with 2 equiv. of **8** in MeOH for 5 days. Compound **10** was subsequently deprotected to generate the phenol derivative (*S*,*S*,*S*)-(*S*,*S*,*S*)-**11**, followed by a [1 + 1] reaction with 1,3,5-tris(bromomethyl)benzene in DMF under high dilution conditions to give the cage (*S*,*S*,*S*)-(*S*,*S*,*S*)-**12**. Compared to our previously reported synthesis of compound **11** based on the deprotection of methoxy groups,^10^ this new pathway gives a higher yield (56% *vs.* 33%) and displays an easier purification step. Finally, the oxido-vanadium complex (*S*,*S*,*S*)-(*S*,*S*,*S*)-**1** was obtained by the reaction of **12** with 1.0 equiv. of vanadium(V)-oxytriisopropoxide in CHCl_3_. Derivative (*S*,*S*,*S*)-(*R*,*R*,*R*)-**12** was obtained following the same procedure starting from enantiopure binaphthol *S*-**6** and (*R*)-(–)-glycidyl nosylate. However, the subsequent complexation of vanadium failed to give the desired complex, even in the presence of an excess of vanadium(V)-oxytriisopropoxide, probably because of the more hindered conformation of the cavity.

### 2.2 Catalytic properties

These complexes were first tested in the oxidation of thioanisol sulfide into sulfoxide, using cumene hydroperoxide (CHP) as the terminal oxidant with 1.5 mol% of catalyst loading in CH_2_Cl_2_ at 0 °C. The results at 180 min and the kinetics are shown in Table 1 and Fig. S1,† respectively. It can be seen that (i) with CHP as the oxidant, all the supramolecular complexes in Set II are very efficient, giving rise to high yields from 85% (*P*-(*S*,*S*,*S*)-(*R*,*R*,*R*)-**2**) to 93% (*P*-(*S*,*S*,*S*)-(*S*,*S*,*S*)-**2**) (Table 1, entries 2–5). The excellent selectivity is (>91%) evidenced by the typical kinetic curves of both yield and conversion of *P*-(*S*,*S*,*S*)-(*S*,*S*,*S*)-**2** which are almost identical (Fig. S2†). (ii) In addition, the yields obtained with hemicryptophane catalysts **2** (entries 2–5) are also significantly higher than that obtained with the corresponding model catalysts **4**, which lack a cavity (entries 8 and 9), and the performances of catalysts **4** and **5** are very close, which indicate that the cage structure is essential for efficient sulfoxidation. (iii) Noticeably, the hemicryptophane catalysts **2** are more effective than the smaller cage catalyst **1** (entry 1), which highlights the important role of the cavity size. (iv) Furthermore, the yields with the new hemicryptophane catalysts in Set II are much higher than those with the previous hemicryptophane catalysts in Set III (entries 6 and 7). The improvement of the hydrophobicity and more pronounced confinement effect induced by these specific bulky binaphthol linkages could account for the enhancement of the reaction rates and yields. (v) It is also interesting to note that the kinetic curves, which monitor the yields as a function of time, are very close to each other among the catalysts in each set (Fig. S1†). The use of *tert*-butyl hydroperoxide (TBHP) as the terminal oxidant led to similar trends in reactivity (Fig. S3†). Therefore, the order of the catalytic activity,† *i.e.* Set II > Set I > Set III > Set IV ≈ Set V, emphasizes that only the cages simultaneously containing CTV, binaphthol and oxido-vanadium moieties can serve as highly efficient supramolecular catalysts. The specific shape of the confined hydrophobic space above the metal center efficiently isolates the substrate from the surrounding solvent and expels the hydrophilic product, thus leading to a strong improvement in activity.^2^ Moreover, the stabilization of the transition state with catalysts **2** may also account for this improvement since the oxygen transfer will give a partial positive charge on the sulphur atom in the transition state which can be better stabilized by the numerous aromatic rings of the cage.

**Table 1:** Oxidation of thioanisol with CHP in the presence of catalyst^*a*^

Entry	Catalyst		Yield^*b*^ (%)	Selectivity^*b*^ (%)
1	Set I	(*S*,*S*,*S*)-(*S*,*S*,*S*)-**1**	74	93
2	Set II	*P*-(*S*,*S*,*S*)-(*S*,*S*,*S*)-**2**	93	97
3		*M*-(*S*,*S*,*S*)-(*S*,*S*,*S*)-**2**	88	94
4		*P*-(*S*,*S*,*S*)-(*R*,*R*,*R*)-**2**	85	91
5		*M*-(*S*,*S*,*S*)-(*R*,*R*,*R*)-**2**	91	97
6	Set III	*M*-(*S*,*S*,*S*)-**3**	40	90
7		*P*-(*S*,*S*,*S*)-**3**	39	90
8	Set IV	(*S*,*S*,*S*)-(*R*,*R*,*R*)-**4**	16	64
9		(*S*,*S*,*S*)-(*S*,*S*,*S*)-**4**	15	50
10	Set V	(*S*,*S*,*S*)-**5**	16	89

^*a*^Conditions: 1.5 mol% catalyst, 1.0 equiv. of CHP, CH_2_Cl_2_, 0 °C, 180 min.

^*b*^Yield and selectivity were determined by HPLC with benzophenone as the internal standard. Selectivity is defined as the yield/conversion ratio.

**Fig. 2:**
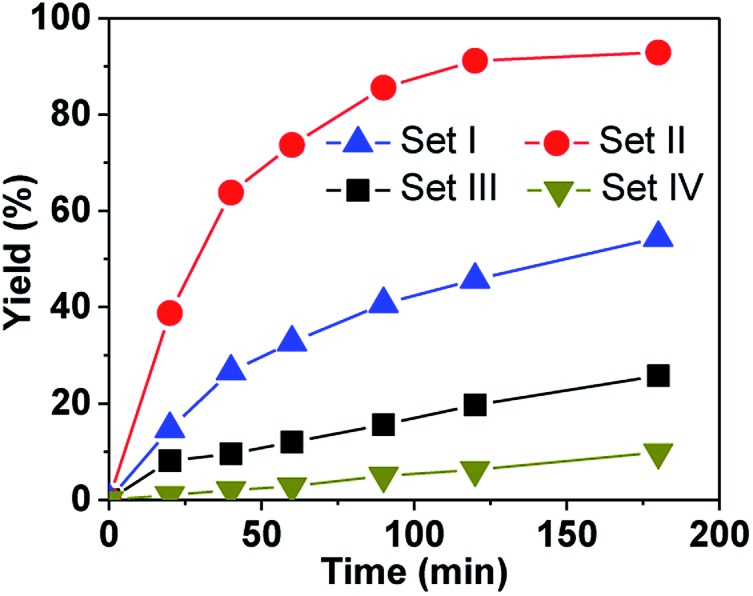
Oxidation of thioanisol with four typical catalysts: (*S*,*S*,*S*)-(*S*,*S*,*S*)-**1** in Set I, *P*-(*S*,*S*,*S*)-(*S*,*S*,*S*)-**2** in Set II, *M*-(*S*,*S*,*S*)-**3** in Set III and (*S*,*S*,*S*)-(*S*,*S*,*S*)-**4** in Set IV (1.0 mol% catalyst, 1.0 equiv. of CHP, 0 °C, CH_2_Cl_2_).

To more accurately evaluate ratio of the kinetic constants, the catalyst amount was decreased to 1.0 mol% while the other conditions were kept identical and CHP used as the oxygen source. As shown in Fig. 2, the reaction rate with *P*-(*S*,*S*,*S*)-(*S*,*S*,*S*)-**2** is much faster than with the other catalysts, and 91% yield was reached within 120 min. Based on the initial rate, the kinetic constant with *P*-(*S*,*S*,*S*)-(*S*,*S*,*S*)-**2** was estimated to be 3-, 5- and 33-fold higher than with (*S*,*S*,*S*)-(*S*,*S*,*S*)-**1**, *M*-(*S*,*S*,*S*)-**3** and (*S*,*S*,*S*)-(*S*,*S*,*S*)-**4**, respectively, which again emphasizes the efficiency of the new hemicryptophane complexes.

**Fig. 3:**
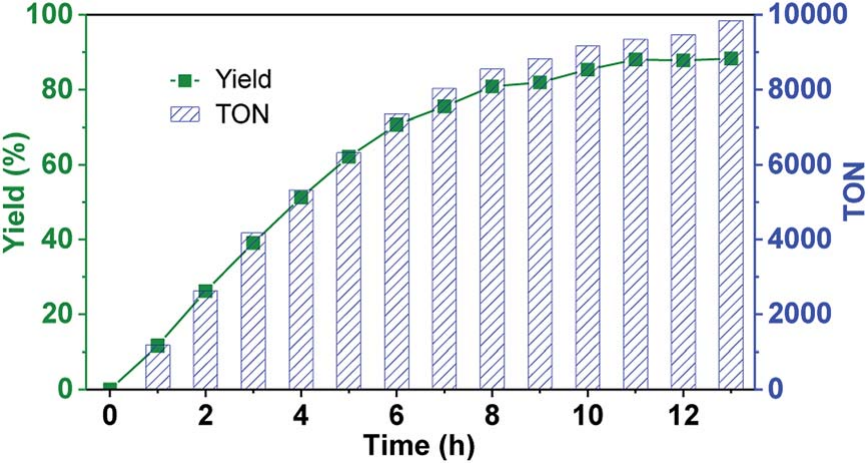
Oxidation of thioanisol catalyzed by catalyst *M*-(*S*,*S*,*S*)-(*R*,*R*,*R*)-**2** (0.01 mol% catalyst, 1.0 equiv. of CHP, r.t., CH_2_Cl_2_).

Since the confinement can strongly improve the reaction rate and no product inhibition effect was exhibited, we wondered if the new hemicryptophane complexes in Set II can overcome the issue of a low TON, which is commonly observed in supramolecular catalysis.^5^ Thus, the oxidation of thioanisol was carried out with *M*-(*S*,*S*,*S*)-(*R*,*R*,*R*)-**2** at a low loading (0.01%) at r.t. As shown in Fig. 3, a yield of 85% was obtained within 10 h, and a rarely observed remarkable TON up to 10 000 was reached after 13 h. The turnover frequency (TOF) of 1200 h^–1^ further indicates the high catalytic activity of this family of confined catalysts. Albeit the catalyst loading was extremely low, the high selectivity for the reaction was also retained (>90%), which suggests the stability of the catalyst. To further evaluate this stability, catalytic cycles using the same catalyst (0.1 mol%) for the oxidation of thioanisol in CH_2_Cl_2_ at room temperature was performed. It was found that no noticeable alteration in catalytic activity over four-cycle experiments was observed (Fig. S4†), which emphasizes the high stability of the encaged catalyst. Obviously, the constraint cage structure protects the metal active site from degradation, which we, and also other groups, have previously reported.^5,7d^

### 2.3 Mechanism investigation

**Fig. 4:**
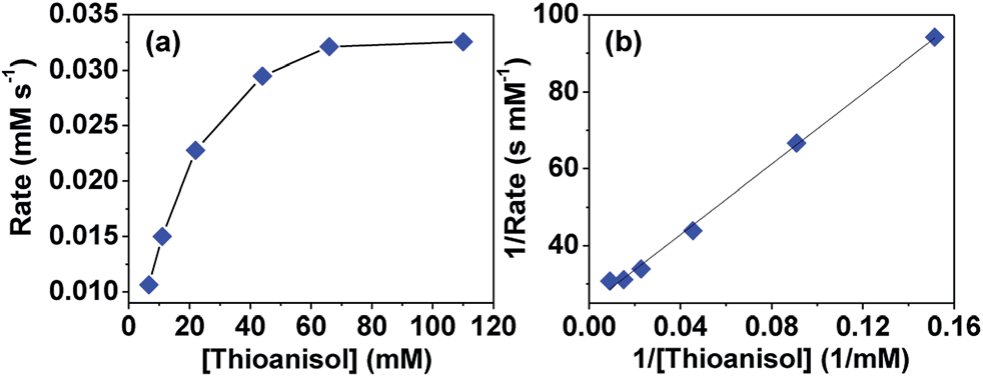
(a) Initial rate dependence on the concentration of thioanisol in CH_2_Cl_2_ with 1.1 mM hemicryptophane catalyst *M*-(*S*,*S*,*S*)-(*R*,*R*,*R*)-**2** and 110 mM CHP at 0 °C. (b) Corresponding Lineweaver–Burke line plotted by 1/rate as a function of 1/[thioanisol].

To understand whether the reaction catalyzed by hemicrytophane catalysts is similar to enzymatic catalysis, more detailed investigations of Michaelis–Menten kinetics were explored. In enzymatic reactions, a substrate and enzyme typically participate in a reversible equilibrium with an enzyme–substrate complex, and usually rate saturation at high substrate concentrations can be observed.^2*g*^ As shown in Fig. 4a, when the substrate concentration was increased while maintaining constant amounts of hemicryptophane catalyst *M*-(*S*,*S*,*S*)-(*R*,*R*,*R*)-**2** and oxidant, initial rate saturation was observed, which is consistent with the Michaelis–Menten rate profile. Strict compliance was further confirmed by re-plotting the data to give the Lineweaver–Burk plot in Fig. 4b with an excellent fit (*R* ^2^ = 0.99). The slope and *y*-intercept gave *K*_m_ = 18.72 mM and *V*_max_ = 4.08 × 10^–2^ mM s^–1^. The latter value was used to calculate the turnover rate, *K*_cat_ (*V*_max_/[catalyst]), which was 3.71 × 10^–2^ s^–1^. To determine the rate enhancement, a *K*_cat_/*K*_uncat_ background experiment, which was oxidation of thioanisol by CHP under the same conditions without any catalyst, was performed. It was observed that after 7 days, the conversion was <2%, thus giving rise to a *K*_uncat_ of <3.3 × 10^–9^ s^–1^. Therefore, it can be evaluated that the rate acceleration, *K*_cat_/*K*_uncat_, is larger than 1.12 × 10^7^, which is an enzyme-like value.

**Fig. 5:**
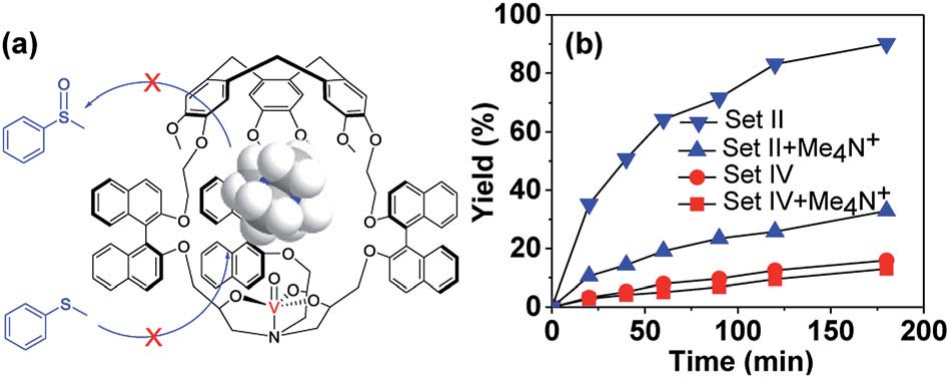
(a) Graphical representation of catalysis inhibition by Me_4_N^+^. (b) Oxidation of thioanisol in the absence or presence of Me_4_N^+^ catalyzed by *M*-(*S*,*S*,*S*)-(*R*,*R*,*R*)-**2** of Set II or (*S*,*S*,*S*)-(*R*,*R*,*R*)-**4** of Set IV (1.5 mol% catalyst, 1.0 equiv. of CHP, 0 °C, CH_2_Cl_2_).

Another characteristic aspect of enzymatic catalysis is the possible inhibition of the enzyme active site with a suitable nonreactive molecule. A bound inhibitor can exclude the substrate from the cavity, thereby inhibiting the activity of the enzyme. The Me_4_N^+^ cation was expected to be encapsulated by the hemicryptophane cage because of the possible cation–π interactions between this cationic guest and numerous aromatic rings of the cage.^11^ As shown in Fig. S5,† upon progressive addition of a concentrated solution of the host *M*-(*S*,*S*,*S*)-(*R*,*R*,*R*)-**2** to a solution of guest Me_4_N^+^Pic^–^ in CD_2_Cl_2_, the ^1^H NMR signals of the protons of Me_4_N^+^ display up-field shifts, which can be attributed to the shielding effect induced by guest encapsulation. Modelling the titration curve with HypNMR2008 afforded a binding constant of 547 M^–1^ for the 1 : 1 host–guest complex (Fig. S5†).^12^ Hence Me_4_N^+^ was considered as the competitive inhibitor, and oxidation of thioanisol in the presence of Me_4_N^+^ catalyzed by either the cage *M*-(*S*,*S*,*S*)-(*R*,*R*,*R*)-**2** or model (*S*,*S*,*S*)-(*R*,*R*,*R*)-**4** catalyst was carried out. Me_4_N^+^Pic^–^ (1.33 catalyst equiv.) was added to the reaction mixture since this concentration corresponds to its solubility limit in CH_2_Cl_2_ at r.t., and also, based on the binding constant, on average more than half of the cavities can be occupied by the Me_4_N^+^ guest. As expected, the catalytic activity of the cage *M*-(*S*,*S*,*S*)-(*R*,*R*,*R*)-**2** strongly decreased in the presence of Me_4_N^+^, whereas that of the model compound (*S*,*S*,*S*)-(*R*,*R*,*R*)-**4** almost remained constant (Fig. 5). Indeed, the reaction rate became 3.5-fold slower in the presence of 1.33 equiv. of Me_4_N^+^ when the cage *M*-(*S*,*S*,*S*)-(*R*,*R*,*R*)-**2** was used as the catalyst, while no obvious change was observed with the open model catalyst. These results evidence the crucial role played by the cavity and also indicate that the catalytic process occurs inside the cavity. Once the cavity is blocked, the benefit induced by the hydrophobic confinement is lost, hence the catalytic activity of the cage complex drops significantly.

**Fig. 6:**
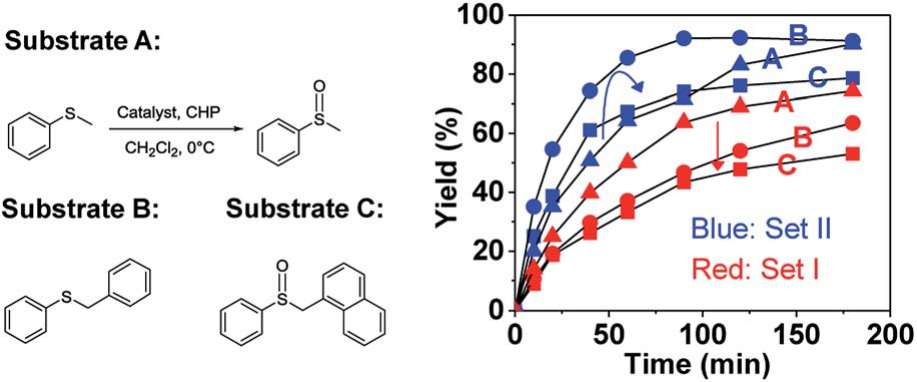
Oxidation of thioanisol (substrate A), benzylphenyl sulfide (substrate B), or naphthylmethyl phenyl sulfide (substrate C) with catalyst *M*-(*S*,*S*,*S*)-(*R*,*R*,*R*)-**2** in Set II or (*S*,*S*,*S*)-(*S*,*S*,*S*)-**1** in Set I (1.5 mol% catalyst, 1.0 equiv. of CHP, 0 °C, CH_2_Cl_2_).

To further elucidate the significant role of the cavity on the catalysis, catalytic oxidation of larger substrates, such as benzylphenyl sulfide (B) and naphthylmethyl phenyl sulfide (C), was performed to explore the substrate size-selectivity (Fig. 6). Under the same conditions used for thioanisol (A), the new hemicryptophane complexes in Set II are still the most efficient catalysts for both substrates (B and C), displaying high yield and selectivity (Fig. S6–S9†). Interestingly, it was found that the reaction rate catalyzed by the smaller cage (*S*,*S*,*S*)-(*S*,*S*,*S*)-**1** continually decreased with an increase in substrate size (Fig. 6). In contrast, the reactivity of *M*-(*S*,*S*,*S*)-(*R*,*R*,*R*)-**2** first increased when the substrate was changed from thioanisol (A) to benzylphenyl sulfide (B), followed by a remarkable drop for the oxidation of the largest naphthylmethyl phenyl sulfide (C) (Fig. 6). Moreover, the Michaelis–Menten kinetics for B and C with catalyst *M*-(*S*,*S*,*S*)-(*R*,*R*,*R*)-**2** were also investigated (Fig. S10 and 11†), and it was found that the values of *K*_cat_ are 3.71 × 10^–2^ s^–1^, 4.89 × 10^–2^ s^–1^ and 3.78 × 10^–2^ s^–1^ for substrates A, B and C, respectively, with a *K*_m_ of 18.72 mM, 17.13 mM and 18.33 mM. The smaller *K*_m_ of substrate B suggests the better binding of B by the cage **2** compared with that of A and C.^3^ Therefore, all these results indicate that benzylphenyl sulfide and naphthylmethyl phenyl sulfide are too sterically demanding to fully access the cavity of (*S*,*S*,*S*)-(*S*,*S*,*S*)-**1**, while the size of benzylphenyl sulfide is suitable and complementary for the cavity of *M*-(*S*,*S*,*S*)-(*R*,*R*,*R*)-**2**. This substrate size-dependent behavior further evidences that the cavity of supramolecular catalysts plays an essential role for efficient catalysis that mimics enzymatic functions.

## 3. Conclusions

In summary, using the oxidation of sulfide as a model reaction, we have described herein five sets of oxido-vanadium(V) complexes, including both cages (Sets I–III) and open (Sets IV and V) structures. It was found that only the complexes (Set II) simultaneously holding CTV, binaphthol and oxido-vanadium groups are the most efficient catalysts because of the construction of a hydrophobic cavity with the right size, which is suitable to accommodate and convert the sulfide substrates, and to expel the product from the inner cavity. The oxidation of thioanisol using the catalysts of Set II displayed reaction rates 3-, 5- and 33-fold faster than that obtained with catalysts of Sets I, III and IV, respectively. The specific shape of the confined hydrophobic space above the metal center induced by the bulky binaphthol linkages, leads to strong improvement in yield, selectivity and also catalytic activity. Hence, the present case constitutes a rare example of supramolecular catalysts that exhibit extremely high levels of efficiency with 10 000 turnovers. The catalytic reaction obeys the Michaelis–Menten model of enzyme kinetics, and competitive inhibition of this reaction was observed using Me_4_N^+^ as an inhibitor. The substrate size-selectivity of the cage catalysts further indicates that the reaction occurs inside the cavity of the hemicryptophane hosts, thus mimicking enzymatic functions. Determination of the catalytic activities of these new supramolecular catalysts toward other reactions, which benefit from the encapsulated vanadium(V) active sites, such as the oxidation of lignin models, are in progress in our laboratory.
