# Supplementary material for: Tailored oxido-vanadium(V) cage complexes for selective sulfoxidation in confined spaces
Source: Chem Sci. 2016 Sep 5;8(1):789–94. doi: 10.1039/c6sc03045a (PMC5299934; doi:10.1039/c6sc03045a)
Supplement: Supplementary file 1 [file SC-008-C6SC03045A-s001.pdf]

Electronic Supplementary Material (ESI) for Chemical Science.
This journal is © The Royal Society of Chemistry 2016

Supporting Information for

Tailored Oxido-Vanadium(V) Cage Complexes for Selective Sulfoxidation in Confined Spaces

Dawei Zhang,[a,b] Kelsey Jamieson,[b] Laure Guy,*[b] Guohua Gao,*[a] Jean-Pierre Dutasta,[b] and Alexandre Martinez*[b,c]

[a] Shanghai Key Laboratory of Green Chemistry and Chemical Processes, School of Chemistry and Molecular Engineering, East China Normal University, 3663 North Zhongshan Road, Shanghai, 200062, P. R. China

[b] Laboratoire de Chimie, École Normale Supérieure de Lyon, CNRS, UCBL, 46 allée d'Italie, F-69364 Lyon, France

[c] Aix Marseille Univ, CNRS, Centrale Marseille, iSm2, Marseille, France

Corresponding Author

laure.guy@ens-lyon.fr; ghgao@chem.ecnu.edu.cn; alexandre.martinez@ens-lyon.fr

Table of Contents

1. Materials and instrumentation 2
2. Synthesis and characterization 2
3. Catalytic properties 12
4. Mechanism investigation 13
5. References 15
6. NMR spectra 16

1. Materials and instrumentation

All solvents used were of commercial grade and were dried prior to use over molecular sieves. ^{1}H NMR and ^{13}C NMR spectra were recorded on a Bruker Avance spectrometer operating at 500.10 MHz and 125.76 MHz for ^{1}H NMR and ^{13}C NMR spectra, respectively. ^{1}H NMR chemical shifts (δ) are reported in ppm and referenced to the protonated residual solvent signal. Mass spectra were recorded by the Centre de Spectrométrie de Masse, Institute of Chemistry, Lyon. HPLC analyses were performed on an Agilent-1100 apparatus (binary pump, autosampler, column, thermostat, and diode array detector) using Chiralpack IC (0.46 × 25 cm) column.

2. Synthesis and characterization

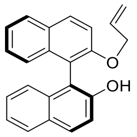

***S*-7/*R*-7:** A solution of K_2CO_3 (3.7 g, 27 mmol) and (*S*)-(-)-1,1'-Bi(2-naphthol) (6.0 g, 20.9 mmol) in acetone (45 mL) was stirred at room temperature for 1 h under argon. Then 5 mL acetone solution of allyl bromide (2.8 g, 23 mmol) was added in one portion. After reflux under argon for 48 h, the solvent was evaporated. The mixture was then dissolved in CH_2Cl_2, and washed with distilled water for 3 times. The organic phase was dried over anhydrous Na_2SO_4, filtrated and evaporated. After column chromatography over silica gel (CH_2Cl_2/petroleum ether 3:2), 4.8 g (70%) *S*-**7** was obtained as a white solid.

^{1}H NMR (CD_2Cl_2, 298K, 500.1 MHz): δ 8.08 (d, *J* = 9.1 Hz, 1H); 7.94 (d, *J* = 8.8 Hz, 2H); 7.90 (d, *J* = 8.1 Hz, 1H); 7.50 (d, *J* = 9.1 Hz, 1H); 7.41 (t, *J* = 7.4 Hz, 1H); 7.36-7.29 (m, 3H); 7.24 (t, *J* = 8.5 Hz, 1H); 7.16 (d, *J* = 8.5 Hz, 1H); 7.05 (d, *J* = 8.4 Hz, 1H); 5.86-5.80 (m, 1H); 5.12-5.08 (m, 2H); 5.03 (s, 1H); 4.63-4.61 (m, 2H).

^{13}C NMR (CD_2Cl_2, 298K, 125.7 MHz): δ 155.0, 151.3, 134.0, 133.8, 133.2, 130.8, 129.7, 129.6, 129.1, 128.2, 128.1, 127.1, 126.4, 124.6(9), 124.6(5), 124.2, 123.2, 117.4, 116.9, 116.0, 115.4, 115.1, 69.8.

ESI-HRMS m/z: found 349.1196, calcd for $C_{23}H_{18}NaO_2$ $[M+Na]^+$ 349.1199.

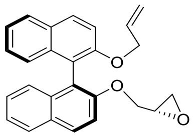

***S-S*-8:** To NaH (131 mg, 3.28 mmol) (60% suspension in oil), a solution of *S*-**7** (622 mg, 1.91 mmol) in DMF (22 mL) was added at room temperature. After stirring under argon for 60 min, a solution of *S*-(+)-glycidyl nosylate (621 mg, 2.40 mmol) in DMF (8 mL) was added. The mixture was stirred at room temperature under argon for 24 h. Then the reaction was quenched by adding saturated NH_4Cl solution and the resulting mixture was diluted with water and extracted with ethyl acetate. The organic phase was washed with water, dried over anhydrous Na_2SO_4, filtrated and evaporated. After column chromatography over silica gel (CH_2Cl_2 as eluent), 730 mg (100 %) *S*-*S*-**8** was obtained as a white solid.

^{1}H NMR (CD_2Cl_2, 298K, 500.1 MHz): δ 8.01 (d, *J* = 9.0 Hz, 2H); 7.92 (d, *J* = 8.2 Hz, 2H); 7.50 (d, *J* = 9.1 Hz, 1H); 7.47 (d, *J* = 9.1 Hz, 1H); 7.36 (q, *J* = 6.7 Hz, 2H); 7.25 (t, *J* = 8.1 Hz, 2H); 7.12 (t, *J* = 8.3 Hz, 2H); 5.85-5.79 (m, 1H); 5.07-5.04 (m, 2H); 4.59-4.58 (m, 2H); 4.26 (dd, *J* = 2.6, 11.6 Hz, 1H); 3.98 (dd, *J* = 5.4, 11.6 Hz, 1H); 3.0 (bs, 1H); 2.59 (t, *J* = 4.7 Hz, 1H); 2.34 (dd, *J* = 2.5, 4.8 Hz, 1H).

^{13}C NMR (CD_2Cl_2, 298K, 125.7 MHz): δ 154.0(3), 153.9(8), 133.9(8), 133.9(5), 133.7, 129.6, 129.4, 129.3(0), 129.2(5), 127.9, 126.3, 125.2, 125.0, 123.8, 123.6, 120.5, 119.6, 116.3, 115.8, 115.3, 70.2, 69.8, 50.2, 44.0.

ESI-HRMS m/z: found 405.1455, calcd for $C_{26}H_{22}NaO_3$ $[M+Na]^+$ 405.1461.

***S-R*-8:** The procedure was similar as that of *S*-*S*-**8** using the starting materials with the corresponding chirality in 90% yield.

^{1}H NMR (CD_2Cl_2, 298K, 500.1 MHz): δ 8.02 (d, *J* = 8.9 Hz, 2H); 7.93 (d, *J* = 8.0 Hz, 2H); 7.50 (t, *J* = 9.0 Hz, 2H); 7.38 (q, *J* = 7.1 Hz, 2H); 7.26 (bs, 2H); 7.16-7.12 (m, 2H); 5.87-5.81 (m, 1H); 5.10-5.06 (m, 2H); 4.61 (bs, 2H); 4.23 (d, *J* = 11.4 Hz, 1H); 4.00 (dd, *J* = 5.3, 11.4 Hz, 1H); 2.99 (bs, 1H); 2.60 (bs, 1H); 2.39 (bs, 1H).

^{13}C NMR (CD_2Cl_2, 298K, 125.7 MHz): δ 154.1, 154.0, 134.0(0), 133.9(8), 133.7, 129.6, 129.4, 129.3(3), 129.3(0), 128.0, 126.3(4), 126.3(1), 125.3, 125.1, 123.9, 123.6, 120.6, 119.7, 116.4,

115.9, 115.3, 70.4, 69.8, 50.1, 44.1.

ESI-HRMS m/z: found 405.1456, calcd for $C_{26}H_{22}NaO_3$ $[M+Na]^+$ 405.1461.

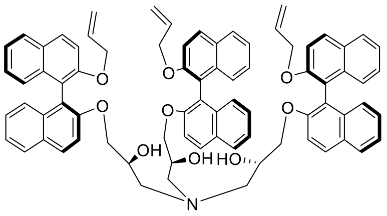

***(S,S,S)*-*(S,S,S)*-10:** To a solution of 7N NH_3 in methanol (75 mL, 525 mmol), (*S,S,S*)-(*S,S,S*)-**8** (450 mg, 1.18 mmol) was added. After stirring at 50 °C for 12 h under argon, the solvent was evaporated. 10 mL pentane was added and evaporated, which was repeated for 3 times to fully remove the residue NH_3. Then 2.0 equiv. of (*S,S,S*)-(*S,S,S*)-**8** (900 mg, 2.35 mmol) and 150 mL MeOH were added under argon. The solution was stirred at 60 °C for 5 days. After evaporation of the solvent, the crude product was purified by column chromatography over silica gel (CH_2Cl_2/MeOH 200:3), 820 mg (60%) (*S,S,S*)-(*S,S,S*)-**10** was obtained as a white solid.

^{1}H NMR (CD_2Cl_2, 298K, 500.1 MHz): δ 8.02 (d, *J* = 9.0 Hz, 3H); 7.93 (d, *J* = 8.1 Hz, 3H); 7.79 (d, *J* = 9.0 Hz, 3H); 7.75 (d, *J* = 8.1 Hz, 3H); 7.45 (d, *J* = 9.0 Hz, 3H); 7.37 (t, *J* = 7.1 Hz, 3H); 7.30 (d, *J* = 9.1 Hz, 3H); 7.26 (q, *J* = 7.6 Hz, 6H); 7.17 (t, *J* = 7.4 Hz, 3H); 7.12 (d, *J* = 8.4 Hz, 3H); 7.04 (d, *J* = 8.5 Hz, 3H); 5.76-5.68 (m, 3H); 4.99-4.95 (m, 6H); 4.51-4.43 (m, 6H); 3.96 (dd, *J* = 3.6, 9.8 Hz, 3H); 3.68 (dd, *J* = 4.4, 9.8 Hz, 3H); 3.00-2.97 (m, 3H); 1.51 (s, 3H); 1.38 (dd, *J* = 2.9, 13.4 Hz, 3H).

^{13}C NMR (CD_2Cl_2, 298K, 125.7 MHz): δ 154.1, 153.8, 133.9(2), 133.8(9), 133.5, 129.5, 129.4, 129.3, 129.2, 128.0, 127.9, 126.3(4), 126.3(2), 125.1(5), 125.0(6), 123.7(3), 123.6(9), 120.0, 119.9, 116.6, 115.7, 115.3, 71.2, 70.0, 66.3, 56.2.

ESI-HRMS m/z: found 1064.5011, calcd for $C_{78}H_{70}NO_9$ $[M+H]^+$ 1164.5045.

***(S,S,S)*-*(R,R,R)*-10:** The procedure was similar as that of (*S,S,S*)-(*S,S,S*)-**10** using the starting materials with the corresponding chirality in 64% yield.

^{1}H NMR (CD_2Cl_2, 298K, 500.1 MHz): δ 8.01 (d, *J* = 9.0 Hz, 3H); 7.93 (d, *J* = 8.2 Hz, 3H); 7.83 (d, *J* = 9.1 Hz, 3H); 7.75 (d, *J* = 8.1 Hz, 3H); 7.44 (d, *J* = 9.1 Hz, 3H); 7.37 (t, *J* = 7.1 Hz, 3H); 7.33

(d, J = 9.0 Hz, 3H); 7.26 (t, J = 7.4 Hz, 3H); 7.21 (t, J = 7.2 Hz, 3H); 7.14-7.05 (m, 9H); 5.78-5.70 (m, 3H); 5.00-4.97 (m, 6H); 4.53-4.45 (m, 6H); 3.79 (d, J = 4.8 Hz, 6H); 3.12-3.10 (m, 3H); 1.70-1.59 (m, 6H).

^{13}C NMR (CD_2Cl_2, 298K, 125.7 MHz): δ 154.1, 153.7, 134.0, 133.9, 133.5, 129.5(2), 129.4(5), 129.3(2), 129.2(9), 128.0, 127.9, 126.4, 126.3, 125.2, 125.1, 123.8, 123.7, 120.2, 119.9, 116.5, 115.7, 115.4, 71.9, 69.9, 66.7, 56.7.

ESI-HRMS m/z: found 1064.5006, calcd for $C_{78}H_{70}NO_9$ $[M+H]^+$ 1164.5045.

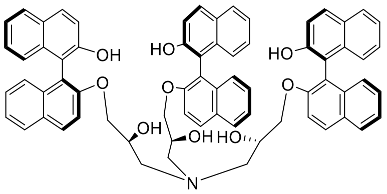

(*S,S,S*)-(*S,S,S*)-11: To a mixture solution of CH_2Cl_2 and MeOH (1/1), (*S,S,S*)-(*S,S,S*)-**10** (800 mg, 0.69 mmol), $Pd(PPh_3)_4$ (794 mg, 0.69 mmol) and K_2CO_3 (950 mg, 6.87 mmol) were added. The mixture was stirred at room temperature for 1 night under argon. After evaporation of the solvent, CH_2Cl_2 and distilled water were added to the mixture. The two layers were then separated and the aqueous phase was extracted with CH_2Cl_2 (1×100 mL), and the organic phase was washed with distilled water (2×50 mL). The combined organic solutions were dried over anhydrous Na_2SO_4, filtrated and evaporated. The crude product was purified by column chromatography on silica gel (first with CH_2Cl_2/MeOH 40/1, then use 200/9) to give (*S,S,S*)-(*S,S,S*)-**11** as a beige solid (402 mg, 56%).

^{1}H NMR (DMSO-d_6, 298K, 500.1 MHz): δ 9.27 (s, 3H); 8.03 (d, J = 9.1 Hz, 3H); 7.94 (d, J = 8.1 Hz, 3H); 7.63 (d, J = 8.5 Hz, 6H); 7.56 (d, J = 9.1 Hz, 3H); 7.34 (t, J = 7.2 Hz, 3H); 7.26-7.22 (m, 6H); 7.16-7.14 (m, 6H); 7.03 (d, J = 8.5 Hz, 3H); 6.87-6.85 (m, 3H); 3.96 (s, 3H); 3.87 (dd, J = 3.3, 9.9 Hz, 3H); 3.57 (dd, J = 3.9, 9.8 Hz, 3H); 2.84 (s, 3H); 1.26 (t, J = 12.7 Hz, 3H); 0.92 (d, J = 12.2 Hz, 3H).

^{13}C NMR (DMSO-d_6, 298K, 125.7 MHz): δ 155.1, 153.1, 134.4, 134.0, 129.6, 129.5, 129.3, 128.4, 128.3, 128.2, 126.6, 126.4, 125.3, 124.6, 123.9, 122.7, 120.2, 118.8, 116.7, 115.7, 72.1, 66.0, 57.4.

ESI-HRMS m/z: found 1044.4064, calcd for $C_{69}H_{58}NO_9$ $[M+H]^+$ 1044.4106.

(*S,S,S*)-(*R,R,R*)-11: The procedure was similar as that of (*S,S,S*)-(*S,S,S*)-**11** using the starting materials with the corresponding chirality in 55% yield.

^{1}H NMR (DMSO-d_6, 298K, 500.1 MHz): δ 9.25 (s, 3H); 8.03 (d, *J* = 9.0 Hz, 3H); 7.94 (d, *J* = 8.1 Hz, 3H); 7.68 (d, *J* = 8.8 Hz, 3H); 7.64-7.62 (m, 3H); 7.54 (d, *J* = 9.0 Hz, 3H); 7.34 (t, *J* = 7.3 Hz, 3H); 7.27-7.23 (m, 6H); 7.04-7.01 (m, 9H); 6.84 (d, *J* = 7.3 Hz, 3H); 4.08 (s, 3H); 3.74-3.64 (m, 6H); 3.03 (s, 3H); 1.42 (t, *J* = 11.3 Hz, 3H); 1.26 (d, *J* = 11.7 Hz, 3H).

^{13}C NMR (DMSO-d_6, 298K, 125.7 MHz): δ 155.1, 153.1, 134.4, 134.0, 129.7, 129.6, 129.3, 128.4, 128.3, 126.6, 126.3, 125.4, 124.6, 124.0, 122.7, 120.5, 118.8, 117.2, 115.6, 72.6, 66.5, 58.1.

ESI-HRMS m/z: found 1044.4054, calcd for $C_{69}H_{58}NO_9$ $[M+H]^+$ 1044.4106.

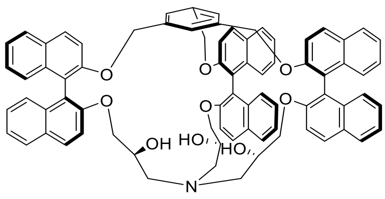

(*S,S,S*)-(*S,S,S*)-12: A solution of Cs_2CO_3 (300 mg, 919 μmol) and (*S,S,S*)-(*S,S,S*)-**11** (160 mg, 153 μmol) in DMF (140 mL) was stirred at 25 ^{o}C for 15 min under argon. Then 140 mL DMF solution of tris(bromomethyl)benzene (60 mg, 169 μmol) was added dropwise. The reaction solution was stirred at 25 ^{o}C for 48 h. Afterwards, 200 mL CH_2Cl_2 was added to the reaction, followed by washing with brine (100×5) to remove DMF and Cs_2CO_3. The organic phase was dried over anhydrous Na_2SO_4, filtrated and evaporated. The crude product was purified by column chromatography on silica gel (first with CH_2Cl_2/MeOH 200/1, then use 200/3) to give (*S,S,S*)-(*S,S,S*)-**12** (53 mg, 30%) as a white solid.

^{1}H NMR (CD_2Cl_2, 298K, 500.1 MHz): δ 8.31 (d, *J* = 9.1 Hz, 3H); 8.24 (d, *J* = 8.8 Hz, 3H); 8.13 (d, *J* = 8.2 Hz, 3H); 8.06 (d, *J* = 8.1 Hz, 3H); 7.73 (d, *J* = 8.8 Hz, 3H); 7.62 (d, *J* = 9.2 Hz, 3H); 7.50-7.47 (m, 3H); 7.39 (t, *J* = 7.3 Hz, 3H); 7.34 (t, *J* = 8.1 Hz, 3H); 7.28 (d, *J* = 8.4 Hz, 3H); 7.21 (t, *J* = 7.6 Hz, 3H); 7.06 (d, *J* = 8.6 Hz, 3H); 5.26 (s, 3H); 4.48 (d, *J* = 10.5 Hz, 3H); 4.30 (d, *J* = 12.2 Hz, 3H); 4.25 (d, *J* = 10.5 Hz, 3H); 3.59 (t, *J* = 10.1 Hz, 3H); 3.38 (bs, 6H); 2.45-2.42

(m, 6H).

^{13}C NMR (CD_2Cl_2, 298K, 125.7 MHz): δ 153.8, 136.1, 134.3, 134.1, 130.6, 129.8, 129.3, 129.2, 128.3, 127.9, 126.8, 126.7, 125.7, 125.5, 124.9, 124.7, 123.9, 123.2, 119.8, 118.1, 114.6, 73.2, 71.5, 70.5, 62.2.

ESI-HRMS m/z: found 1158.4547, calcd for $C_{78}H_{64}NO_9$ $[M+H]^+$ 1158.4576.

(*S,S,S*)-(*R,R,R*)-12: The procedure was similar as that of (*S,S,S*)-(*S,S,S*)-**12** using the starting materials with the corresponding chirality in 43% yield.

^{1}H NMR (CD_2Cl_2, 298K, 500.1 MHz): δ 8.31 (d, *J* = 9.0 Hz, 3H); 8.24 (d, *J* = 9.0 Hz, 3H); 8.06 (t, *J* = 8.7 Hz, 6H); 7.78 (d, *J* = 9.1 Hz, 3H); 7.62 (d, *J* = 9.0 Hz, 3H); 7.48-7.43 (m, 6H); 7.30 (q, *J* = 8.7 Hz, 6H); 7.15 (d, *J* = 8.6 Hz, 3H); 7.12 (d, *J* = 8.5 Hz, 3H); 6.44 (s, 3H); 6.22 (s, 3H); 4.85 (d, *J* = 11.9 Hz, 3H); 4.61 (d, *J* = 11.9 Hz, 3H); 4.21 (dd, *J* = 4.1, 10.5 Hz, 3H); 4.08-4.07 (m, 3H); 3.83-3.80 (m, 3H); 2.65 (t, *J* = 12.7 Hz, 3H); 2.15 (d, *J* = 12.7 Hz, 3H).

^{13}C NMR (CD_2Cl_2, 298K, 125.7 MHz): δ 153.7, 153.4, 137.5, 134.3, 134.2, 129.9(3), 129.8(9), 129.8, 128.1(1), 128.0(6), 126.9, 126.7, 125.4, 125.2, 124.5, 124.3, 121.2, 120.7, 116.0, 115.6, 72.2, 71.1, 66.6, 64.3.

ESI-HRMS m/z: found 1158.4543, calcd for $C_{78}H_{64}NO_9$ $[M+H]^+$ 1158.4576.

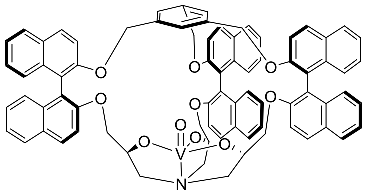

(*S,S,S*)-(*S,S,S*)-1: To 15 mL $CHCl_3$, (*S,S,S*)-(*S,S,S*)-**12** (70 mg, 60.4 µmol) was added. The solution was stirred at room temperature for 5 min. Then vanadium(V) oxytriisopropoxide (14.2 µL, 60.4 µmol) was added to the solution, which was further stirred for 1 h. After evaporation of the solvent, the mixture was dissolved in 10 mL CH_2Cl_2. The solution was filtered by syringe filter (pore size: 0.45 µm), followed by evaporation to give the complex (*S,S,S*)-(*S,S,S*)-**1** (67 mg, 91%) as a dark green solid.

^{1}H NMR (CD_2Cl_2, 298K, 500.1 MHz): δ 8.24 (t, *J* = 8.3 Hz, 6H); 8.17 (d, *J* = 8.2 Hz, 3H); 8.04 (d, *J* = 8.1 Hz, 3H); 7.60 (d, *J* = 8.9 Hz, 3H); 7.54 (d, *J* = 9.2 Hz, 3H); 7.48-7.44 (m, 6H); 7.33-7.27

(m, 6H); 7.18-7.13 (m, 6H); 5.57 (s, 3H); 4.44 (q, *J* = 12.6 Hz, 6H); 4.36-4.35 (m, 3H); 4.16 (t, *J* = 11.1 Hz, 3H); 3.89 (dd, *J* = 4.3, 11.5 Hz, 3H); 2.09 (dd, *J* = 3.6, 12.8 Hz, 3H); 1.83 (t, *J* = 11.5 Hz, 3H).

^{13}C NMR (CD_2Cl_2, 298K, 125.7 MHz): δ 154.0, 153.3, 136.8, 134.4, 134.0, 129.9, 129.8, 129.5, 129.3, 128.1, 127.9, 127.0, 126.8, 126.4, 125.2, 125.1, 124.4(2), 124.3(6), 121.4, 120.7, 116.5, 114.2, 83.1, 71.6, 69.3, 57.1.

ESI-HRMS m/z: found 1244.3526, calcd for $C_{78}H_{60}NNaO_{10}V$ $[M+Na]^+$ 1244.3549.

***(S,S,S)*-*(R,R,R)*-1:** A similar procedure as that of (*S,S,S*)-(*S,S,S*)-**1** has been used for the synthesis of (*S,S,S*)-(*R,R,S*)-**1** using the corresponding ligand. However, the reaction failed to give the desired complex as the ^{1}H NMR spectrum is too complicated to analyze. The different behavior from the synthesis of (*S,S,S*)-(*S,S,S*)-**1** probably because of its more hindered conformation of the cavity.

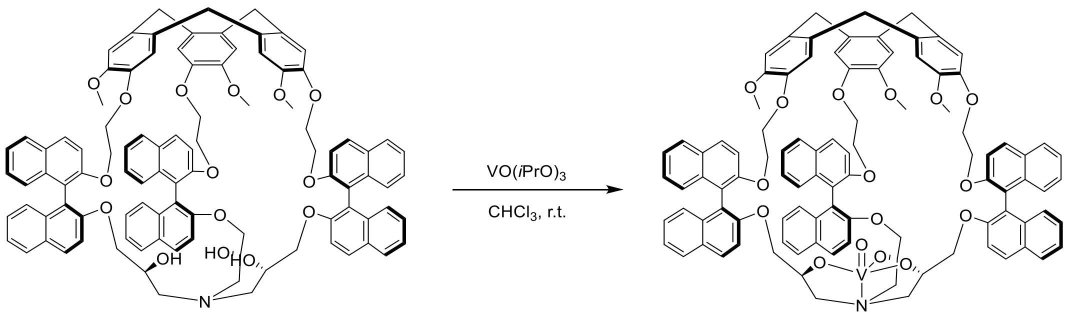

Scheme S1 Synthesis of the oxido-vanadium(V) hemicryptophane complexes **2**.

***P*-*(S,S,S)*-*(S,S,S)*-2:** The ligand of *P*-(*S,S,S*)-(*S,S,S*)-**2** complex was synthesized according to our previous procedure.[1] To 3 mL $CHCl_3$, the ligand of *P*-(*S,S,S*)-(*S,S,S*)-**2** (10 mg, 6.5 µmol) was added. The solution was stirred at room temperature for 5 min. Then vanadium(V) oxytriisopropoxide (4.8 µL, 19.6 µmol) was added to the solution, which was further stirred for 1 h. After evaporation of the solvent, the mixture was dissolved in 5 mL CH_2Cl_2. The solution was filtered by syringe filter (pore size: 0.45 µm), followed by evaporation to give the complex *P*-(*S,S,S*)-(*S,S,S*)-**2** (10 mg, 96%) as a dark green solid.

^{1}H NMR (C_6D_6, 298K, 500.1 MHz): δ 8.42 (d, *J* = 9.2 Hz, 3H); 7.84-7.78 (m 12H); 7.50 (d, *J* = 8.5 Hz, 3H); 7.31-7.17 (m, 12H); 7.02-6.98 (m, 6H); 6.60 (s, 3H); 6.57 (s, 3H); 4.70 (d, *J* = 13.7 Hz, 3H); 4.23 (t, *J* = 11.2 Hz, 3H); 4.02 (t, *J* = 10.2 Hz, 3H); 3.78-3.75 (m, 6H); 3.45-3.40 (m,

9H); 3.04-2.98 (m, 12H); 0.72 (dd, *J* = 3.8, 13.3 Hz, 3H); 0.23 (t, *J* = 12.6 Hz, 3H).

^{13}C NMR (C_6D_6, 298K, 125.7 MHz): δ 156.3, 154.3, 147.7, 146.6, 134.4, 133.8, 131.8, 131.2, 130.2, 130.0, 129.5, 129.2, 128.2, 127.2, 126.5, 126.3, 125.7, 125.3, 124.4, 123.8, 121.1, 121.0, 119.9, 116.1, 112.3, 111.9, 81.4, 72.5, 70.0, 67.2, 56.0, 53.8, 36.7.

ESI-HRMS m/z: found 1617.5128, calcd for $C_{99}H_{84}NNaO_{16}V$ $[M+Na]^+$ 1616.5122.

***M*-(*S,S,S*)-(*S,S,S*)-2, *P*-(*S,S,S*)-(*R,R,R*)-2 and *M*-(*S,S,S*)-(*R,R,R*)-2:** The procedure for each of the complexes from their corresponding ligands was similar as that of *P*-(*S,S,S*)-(*S,S,S*)-**2** except that only 1.0 equiv. of vanadium(V) oxytriisopropoxide instead of 3 equivalents was used. The reason for this difference is because the ligand of *P*-(*S,S,S*)-(*S,S,S*)-**2** shows an extremely imploded conformation, which suffers from a slow vanadium chemical complexation kinetics. For all the complexes, the yields are higher than 90%.

***M*-(*S,S,S*)-(*S,S,S*)-2:**

^{1}H NMR (C_6D_6, 298K, 500.1 MHz): δ 8.13 (d, *J* = 9.1 Hz, 3H); 7.73 (d, *J* = 9.1 Hz, 3H); 7.69 (d, *J* = 9.1 Hz, 3H); 7.63 (dd, J = 8.2, 13.2 Hz, 6H); 7.50 (d, *J* = 9.1 Hz, 3H); 7.26 (d, *J* = 8.5 Hz, 3H); 7.19 (d, *J* = 8.6 Hz, 3H); 7.10 (d, *J* = 7.1 Hz, 3H); 7.05-6.98 (m, 6H); 6.88 (t, *J* = 8.2 Hz, 3H); 6.75 (s, 3H); 6.23 (s, 3H); 4.37 (d, *J* = 13.6 Hz, 6H); 4.06-4.02 (m, 3H); 3.94-3.91 (m, 3H); 3.80-3.77 (m, 3H); 3.70-3.66 (m, 3H); 3.59-3.53 (m, 6H); 3.20 (d, *J* = 13.6 Hz, 3H); 2.40 (s, 9H); 1.94 (t, *J* = 12.2 Hz, 3H); 0.90 (dd, *J* = 3.7, 12.9 HZ, 3H).

^{13}C NMR (C_6D_6, 298K, 125.7 MHz): δ 155.4, 153.4, 148.8, 146.0, 134.5, 133.7, 133.6, 131.7, 130.3, 130.1, 129.6, 129.0, 128.1, 127.8, 126.9, 126.3, 125.7, 125.2, 124.4, 124.1, 122.0, 121.0, 119.4, 118.3, 116.8, 112.7, 83.7, 73.9, 69.1, 67.3, 54.3, 54.2, 36.4.

ESI-HRMS m/z: found 1616.5109, calcd for $C_{99}H_{84}NNaO_{16}V$ $[M+Na]^+$ 1616.5122.

***P*-(*S,S,S*)-(*R,R,R*)-2:**

^{1}H NMR (C_6D_6, 298K, 500.1 MHz): δ 7.95 (d, *J* = 8.4 Hz, 3H); 7.78 (d, *J* = 8.1 Hz, 3H); 7.74 (d, *J* = 9.0 Hz, 3H); 7.65 (d, *J* = 8.2 Hz, 3H); 7.39-7.34 (m, 6H); 7.30-7.28 (m, 6H); 7.21 (d, *J* = 8.5 Hz, 3H); 7.07 (t, *J* = 7.7 Hz, 3H); 7.03 (d, *J* = 9.0 Hz, 3H); 6.93 (t, *J* = 8.0 Hz, 3H); 6.59(2) (s, 3H); 6.58(9) (s, 3H); 4.66 (d, *J* = 13.7 Hz, 3H); 4.24 (dd, J = 5.7, 12.1 Hz, 3H); 3.92-3.84 (m,

6H); 3.78-3.70 (m, 9H); 3.37 (d, *J* = 13.8 Hz, 3H); 3.25-3.20 (m, 3H); 3.06 (s, 9H); 1.04 (dd, *J* = 3.7, 13.2 HZ, 3H); 0.79 (dd, *J* = 10.6, 12.7 HZ, 3H).

^{13}C NMR ($CDCl_3$, 298K, 125.7 MHz): δ 155.3, 153.6, 148.3, 147.0, 133.9, 133.5, 132.0, 131.7, 129.6, 129.3, 128.8, 128.0, 126.8, 126.6, 126.5, 126.3, 125.2, 124.7, 123.8, 121.7, 119.9, 119.7, 114.5, 114.0, 113.7, 81.6, 71.0, 69.6, 68.9, 56.5, 54.6, 36.3.

ESI-HRMS m/z: found 1616.5066, calcd for $C_{99}H_{84}NNaO_{16}V$ $[M+Na]^+$ 1616.5122.

***M*-(*S,S,S*)-(*R,R,R*)-2:**

^{1}H NMR ($CDCl_3$, 298K, 500.1 MHz): δ 8.03 (d, *J* = 9.0 Hz, 3H); 7.97 (d, *J* = 8.1 Hz, 3H); 7.93 (d, *J* = 8.2 Hz, 3H); 7.51 (d, *J* = 9.1 Hz, 3H); 7.46-7.43 (m, 6H); 7.35-7.33 (m, 6H); 7.21-7.15 (m, 6H); 7.10 (s, 3H); 7.01 (d, *J* = 8.5 Hz, 3H); 6.92 (s, 3H); 6.81 (d, *J* = 8.9 Hz, 3H); 4.95 (d, *J* = 13.8 Hz, 3H); 4.50-4.45 (m, 3H); 4.21-4.17 (m, 9H); 3.70-3.67 (m, 6H); 3.38-3.35 (m, 12H); 3.05-3.04 (m, 3H); 0.95 (dd, *J* = 3.8, 13.1 HZ, 3H); 0.62 (t, *J* = 11.3 Hz, 3H).

^{13}C NMR ($CDCl_3$, 298K, 125.7 MHz): δ 154.6, 153.3, 148.7, 145.9, 134.3, 133.9, 133.4, 132.0, 130.3, 130.1, 129.9, 129.6, 128.8, 128.4, 126.9, 126.6, 125.3, 125.0, 124.7, 124.3, 123.2, 122.0, 118.7, 118.6, 116.5, 113.0, 81.9, 72.4, 69.0, 66.7, 55.1, 54.4, 36.7.

ESI-HRMS m/z: found 1616.5090, calcd for $C_{99}H_{84}NNaO_{16}V$ $[M+Na]^+$ 1616.5122.

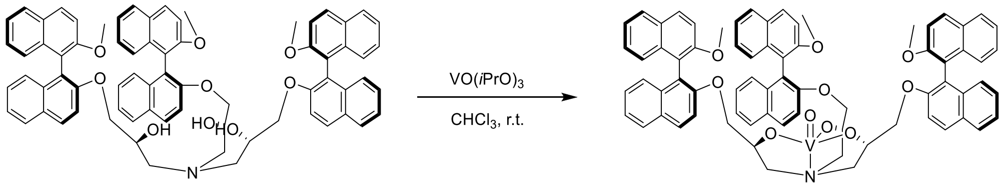

Scheme S2 Synthesis of the oxido-vanadium(V) complexes **4**.

(*S,S,S*)-(*S,S,S*)-4: The ligand of (*S,S,S*)-(*S,S,S*)-**4** complex was synthesized according to our previous procedure.[1] To 30 mL $CHCl_3$, the ligand of (*S,S,S*)-(*S,S,S*)-**4** (300 mg, 276 µmol) was added. The solution was stirred at room temperature for 5 min. Then vanadium(V) oxytriisopropoxide (64.8 µL, 276 µmol) was added to the solution, which was further stirred for 1 h. After evaporation of the solvent, the mixture was dissolved in 15 mL CH_2Cl_2. The solution was filtered by syringe filter (pore size: 0.45 µm), followed by evaporation to give the complex (*S,S,S*)-(*S,S,S*)-**4** (318 mg, 100%) as a yellow solid.

^{1}**H NMR** (CD_2Cl_2, 298K, 500.1 MHz): δ 8.06 (d, *J* = 9.0 Hz, 3H); 7.94 (d, *J* = 8.2 Hz, 3H); 7.82 (d, *J* = 9.1 Hz, 3H); 7.74 (d, *J* = 8.1 Hz, 3H); 7.49 (d, *J* = 9.0 Hz, 3H); 7.41-7.36 (m, 6H); 7.30-7.23 (m, 9H); 7.11 (d, *J* = 8.4 Hz, 3H); 7.06 (d, *J* = 8.4 Hz, 3H); 4.07 (dd, J = 3.2, 9.8 Hz, 3H); 3.74 (s, 9H); 3.65-3.59 (m, 3H); 3.53-3.52 (m, 3H); 1.48 (t, *J* = 12.1 Hz, 3H); 1.24 (dd, *J* = 3.5, 12.8 HZ, 3H).

^{13}**C NMR** (CD_2Cl_2, 298K, 125.7 MHz): δ 155.2, 153.9, 133.9, 133.8, 129.7, 129.6, 129.0, 128.9, 128.0, 127.8, 126.5, 126.4, 125.0, 124.9, 124.0, 123.5, 120.4, 119.0, 115.7, 114.1, 82.2, 70.3, 56.6, 54.1.

ESI-HRMS m/z: found 1172.3518, calcd for $C_{72}H_{60}NNaO_{10}V$ $[M+Na]^+$ 1172.3549.

(*S,S,S*)-(*R,R,R*)-4: The procedure was similar as that of (*S,S,S*)-(*S,S,S*)-**4** using the corresponding ligand in 94% yield.

^{1}**H NMR** (CD_2Cl_2, 298K, 500.1 MHz): δ 8.03 (d, *J* = 9.0 Hz, 3H); 7.95 (t, *J* = 9.3 Hz, 6H); 7.76 (d, *J* = 8.1 Hz, 3H); 7.49 (d, *J* = 9.1 Hz, 3H); 7.46 (d, *J* = 9.0 Hz, 3H); 7.40 (t, *J* = 7.3 Hz, 3H); 7.28 (t, *J* = 7.9 Hz, 3H); 7.21-7.13 (m, 9H); 7.05 (d, *J* = 8.4 Hz, 3H); 3.87 (s, 9H); 3.74-3.67 (m, 6H); 3.64-3.61 (m, 3H); 1.46 (t, *J* = 12.5 Hz, 3H); 1.37 (dd, *J* = 3.8, 12.8 HZ, 3H).

^{13}**C NMR** (CD_2Cl_2, 298K, 125.7 MHz): δ 154.7, 154.0, 134.1, 133.8, 130.0, 129.7, 129.5, 129.0, 128.1, 127.8, 126.6, 126.5, 125.2, 125.1, 124.2, 123.7, 121.1, 119.2, 116.9, 113.4, 82.1, 71.3, 56.4, 54.9.

ESI-HRMS m/z: found 1172.3535, calcd for $C_{72}H_{60}NNaO_{10}V$ $[M+Na]^+$ 1172.3549.

3. Catalytic properties

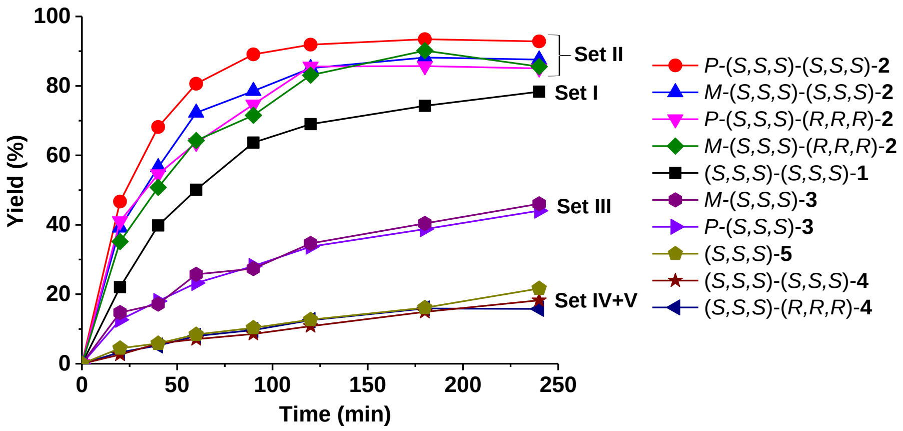

Figure S1 Time course of oxidation of thioanisol with catalysts in different sets (conditions: 1.5 mol% catalyst, 1.0 equiv. of CHP, 0 °C, CH_2Cl_2).

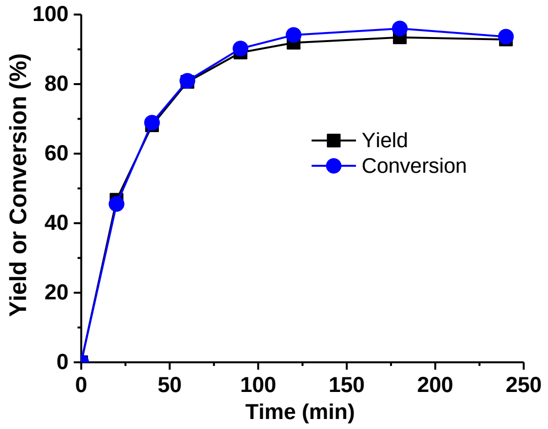

Figure S2 Time course of oxidation of thioanisol with hemicryptophane catalyst *P*-(*S*,*S*,*S*)-(*S*,*S*,*S*)-**2** (conditions: 1.5 mol% catalyst, 1.0 equiv. of CHP, 0 °C, CH_2Cl_2).

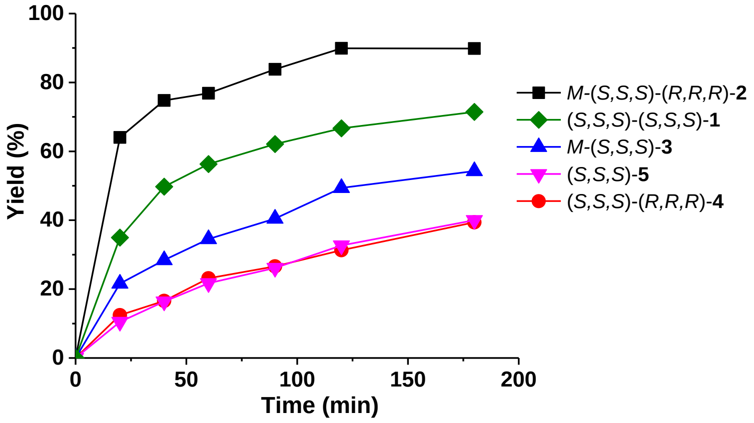

Figure S3 Time course of oxidation of thioanisol with typical catalysts in different sets (conditions: 1.5 mol% catalyst, 1.0 equiv. of TBHP, 0 °C, CH_2Cl_2).

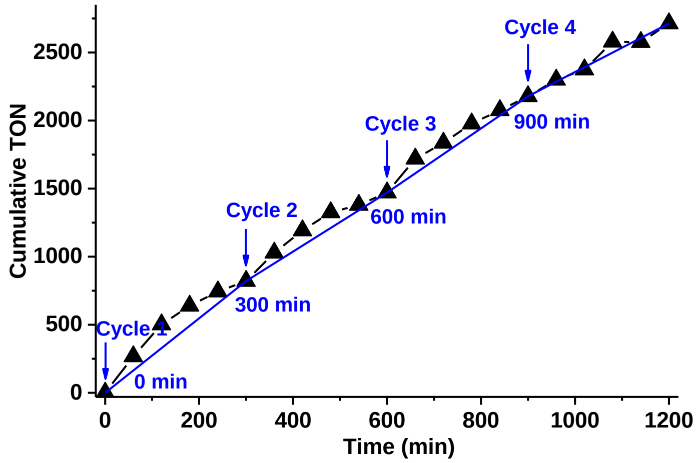

Figure S4 Changes of the cumulative TON over four-cycle experiments catalyzed by *M*-(*S*,*S*,S)-(*R*,*R*,*R*)-**2** with the development of time (conditions: 0.1 mol% catalyst, 1.0 equiv. of CHP, r.t., CH_2Cl_2; after each cycle, 1.0 equiv. of reaction substrate and CHP were reloaded; TON was calculated from the yield).

4. Mechanism investigation

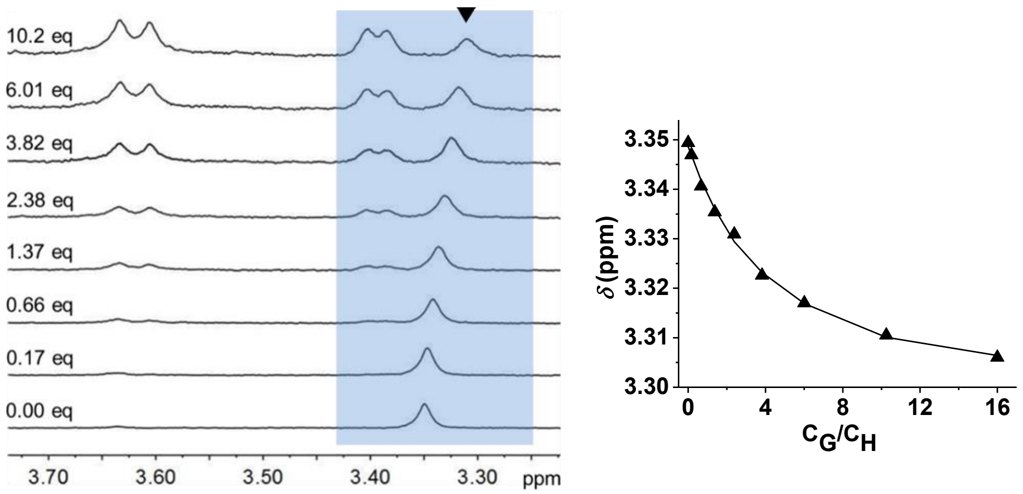

Figure S5 ^{1}H NMR spectra (500 MHz, 298 K) of $Me_4N^+Pic^-$ (▼) in CD_2Cl_2 upon progressive addition of different equivalents of the host *M*-(*S*,*S*,S)-(*R*,*R*,*R*)-**2** (left side) and its titration curve fitted by HypNMR2008 (right side).

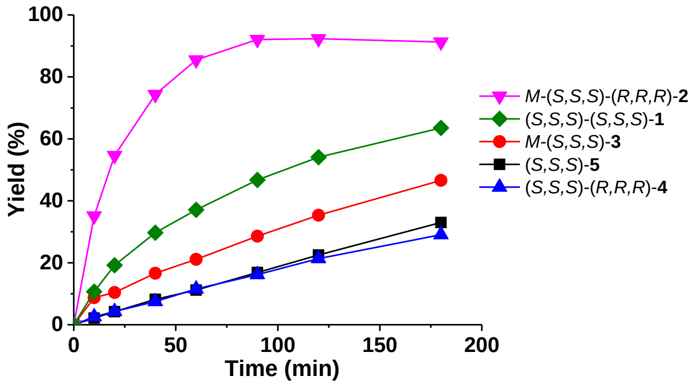

Figure S6 Time course of oxidation of benzylphenyl sulfide with typical catalysts (conditions: 1.5 mol% catalyst, 1.0 equiv. of CHP, 0 ^{o}C, CH_2Cl_2).

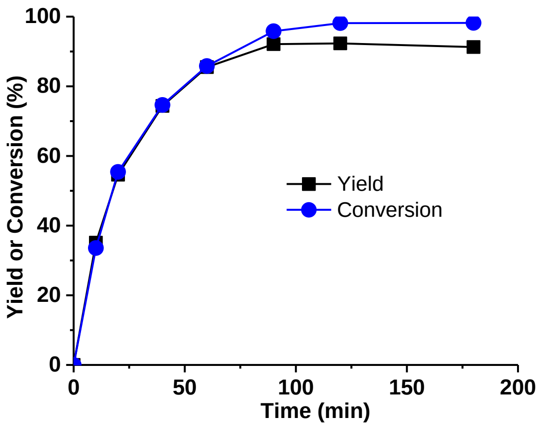

Figure S7 Time course of oxidation of benzylphenyl sulfide with hemicryptophane catalyst *M*-(*S*,*S*,S)-(*R*,*R*,*R*)-**2** (conditions: 1.5 mol% catalyst, 1.0 equiv. of CHP, 0 ^{o}C, CH_2Cl_2).

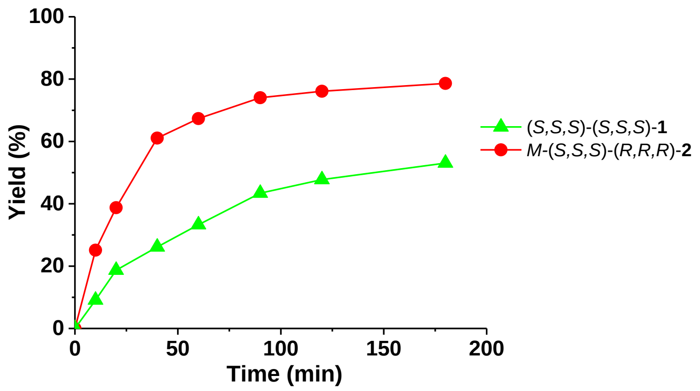

Figure S8 Time course of oxidation of naphthylmethyl phenyl sulfide with typical catalysts (conditions: 1.5 mol% catalyst, 1.0 equiv. of CHP, 0 ^{o}C, CH_2Cl_2).

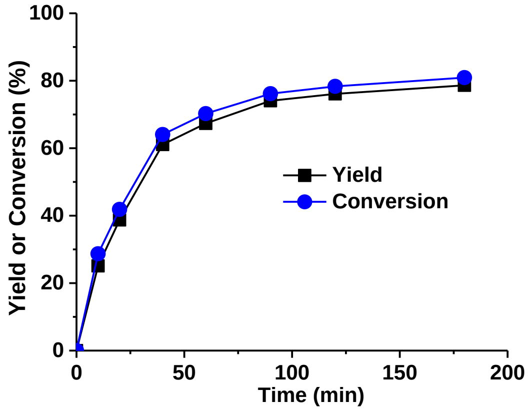

Figure S9 Time course of oxidation of naphthylmethyl phenyl sulfide with hemicryptophane catalyst *M*-(*S*,*S*,S)-(*R*,*R*,*R*)-**2** (conditions: 1.5 mol% catalyst, 1.0 equiv. of CHP, 0 ^{o}C, CH_2Cl_2).

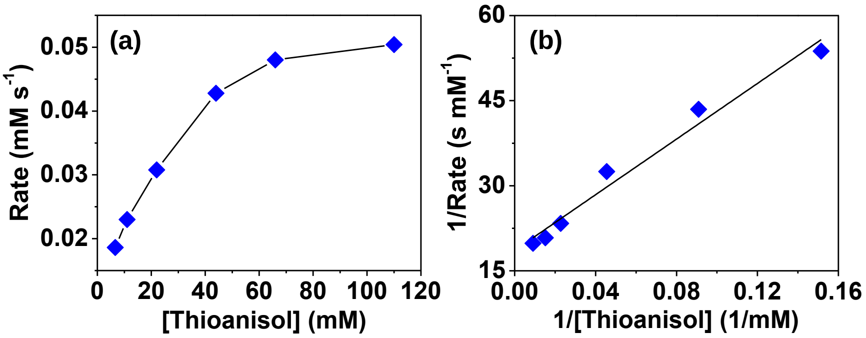

Figure S10 (a) Initial rate dependence on the concentration of benzylphenyl sulfide in CH_2Cl_2 with 1.1 mM hemicryptophane catalyst *M*-(*S*,*S*,*S*)-(*R*,*R*,*R*)-**2** and 110 mM CHP at 0 ^{o}C. (b) The corresponding Lineweaver-Burke line plotted by 1/rate as a function of 1/[benzylphenyl sulfide].

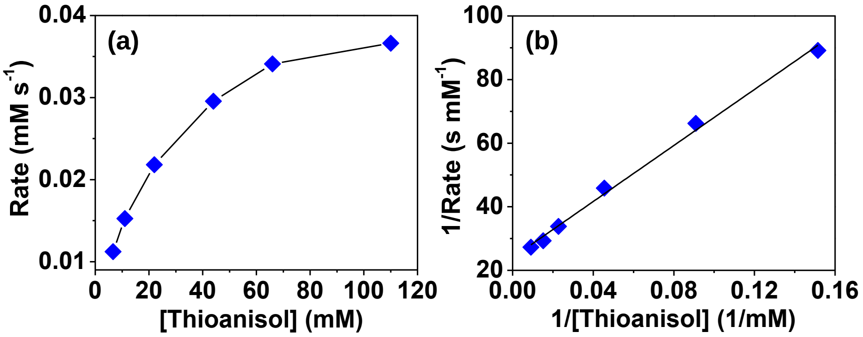

Figure S11 (a) Initial rate dependence on the concentration of naphthylmethyl phenyl sulfide in CH_2Cl_2 with 1.1 mM catalyst *M*-(*S*,*S*,*S*)-(*R*,*R*,*R*)-**2** and 110 mM CHP at 0 ^{o}C. (b) The corresponding Lineweaver-Burke line plotted by 1/rate as a function of 1/[naphthylmethyl phenyl sulfide].

5. References

[1] Zhang, D.; Mulatier, J.-C.; Cochrane, J. R.; Guy, L.; Gao, G.; Dutasta, J.-P.; Martinez, A. *Chem. Eur. J.*, 2016, **22**, 8038.

6. NMR spectra

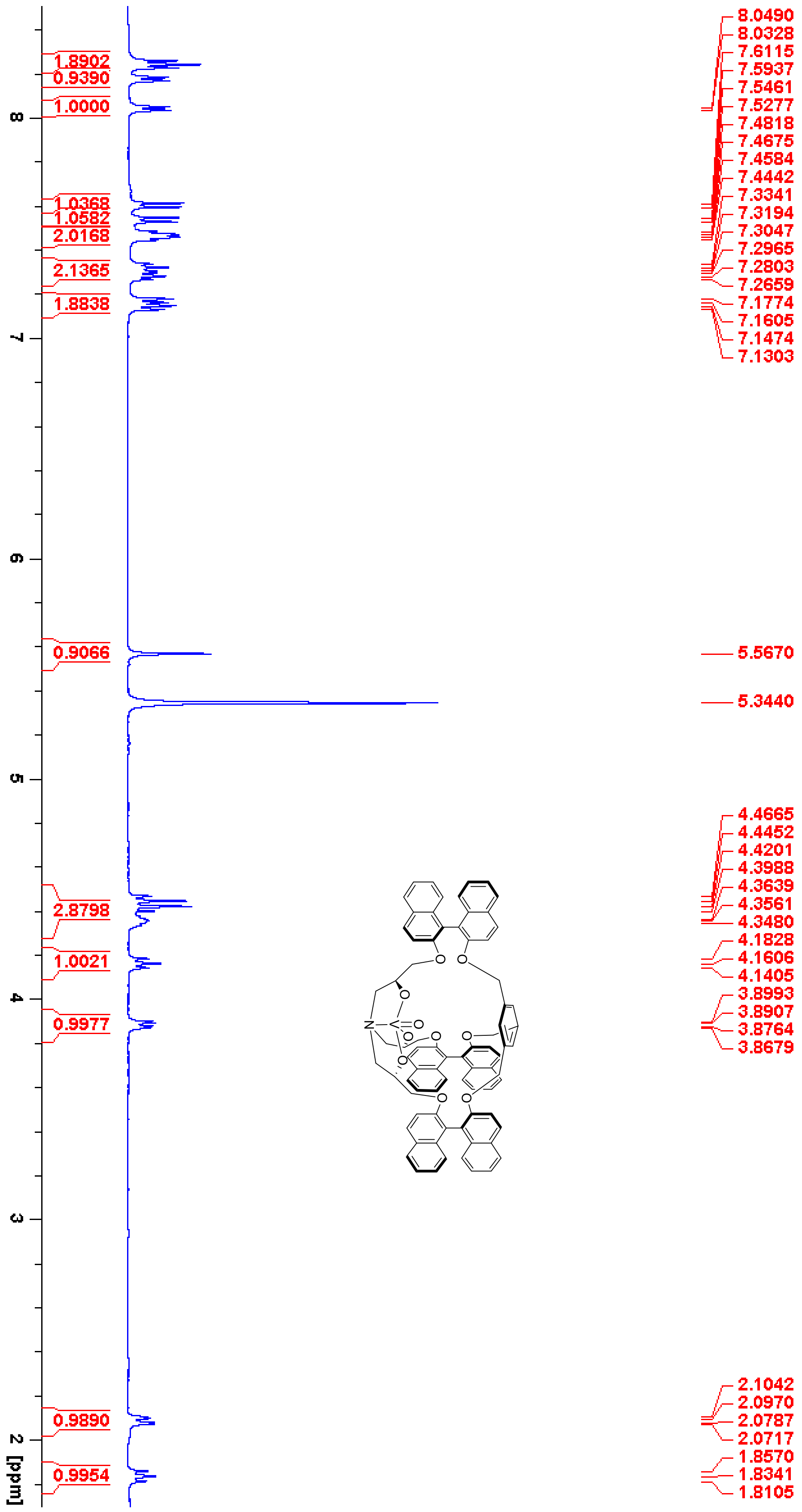

^{1}H NMR spectrum (CD_2Cl_2, 500.1 MHz, 298K) of (S,S,S)-(S,S,S)-**1**.

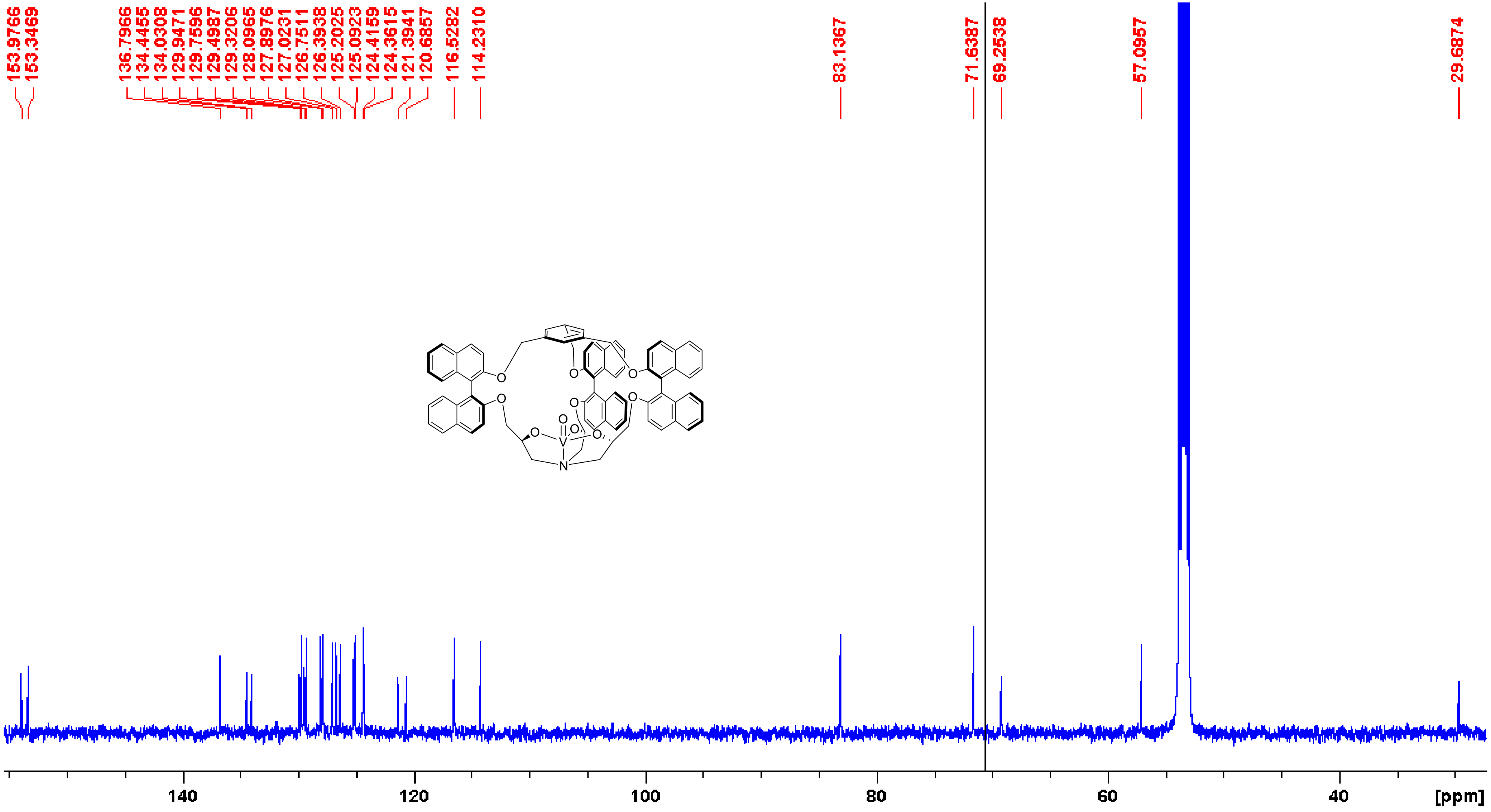

^{13}C NMR spectrum (CD_2Cl_2, 125.8 MHz, 298K) of (*S*,*S*,*S*)-(*S*,*S*,*S*)-**1**.

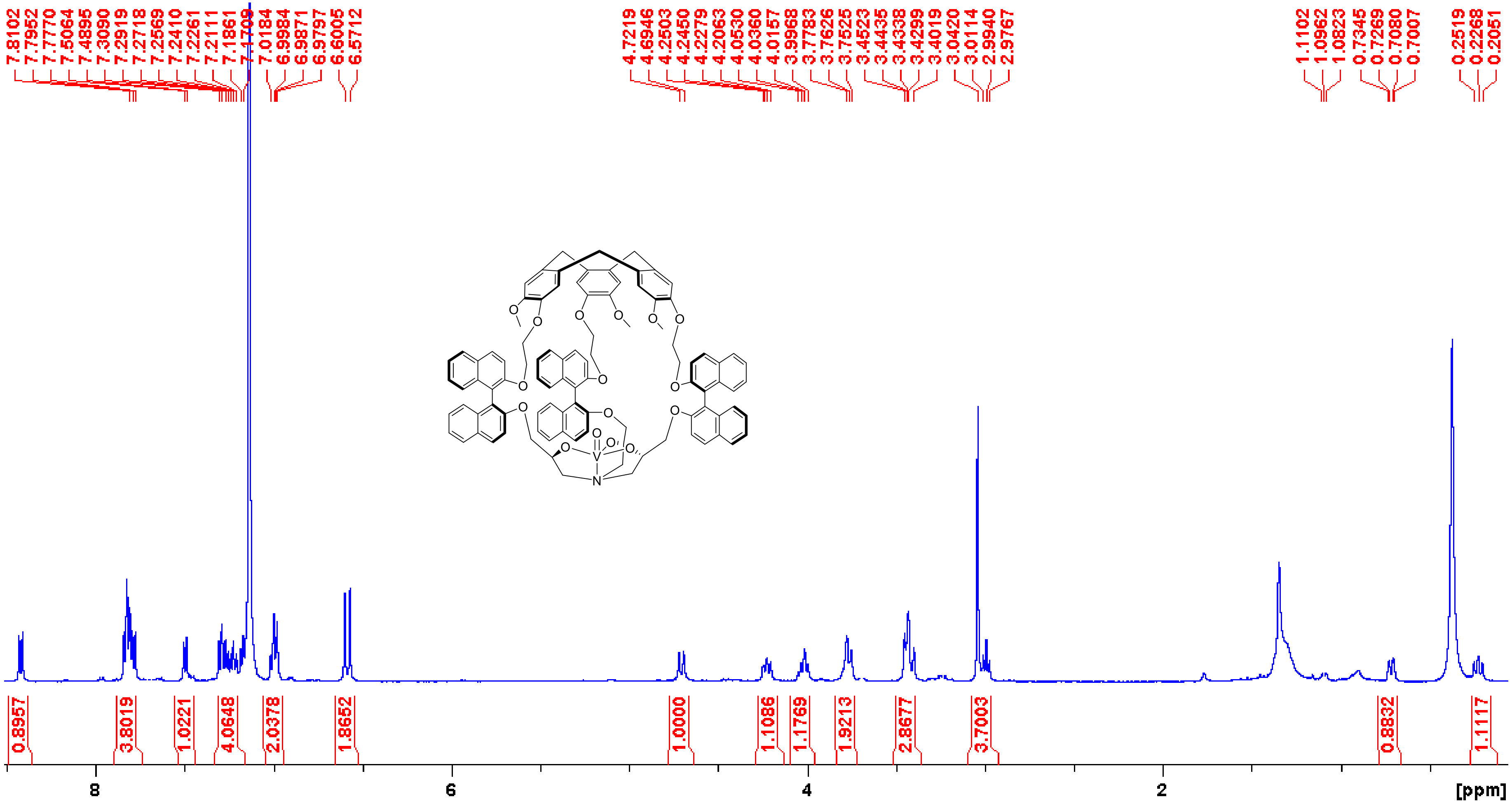

^{1}H NMR spectrum (C_6D_6, 500.1 MHz, 298K) of *P*-(*S*,*S*,*S*)-(*S*,*S*,*S*)-**2**.

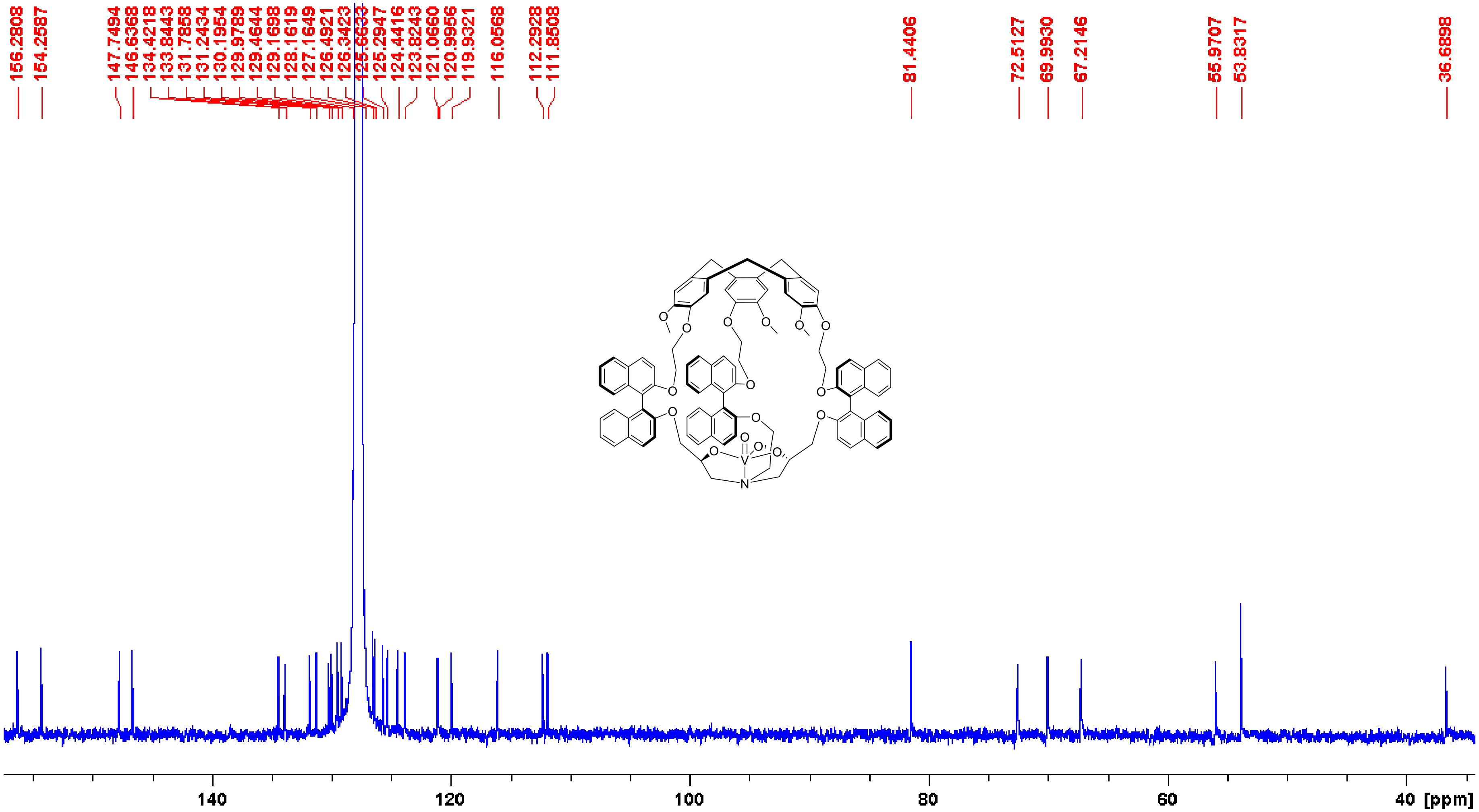

^{13}C NMR spectrum (C_6D_6, 125.8 MHz, 298K) of *P*-(*S*,*S*,*S*)-(*S*,*S*,*S*)-**2**.

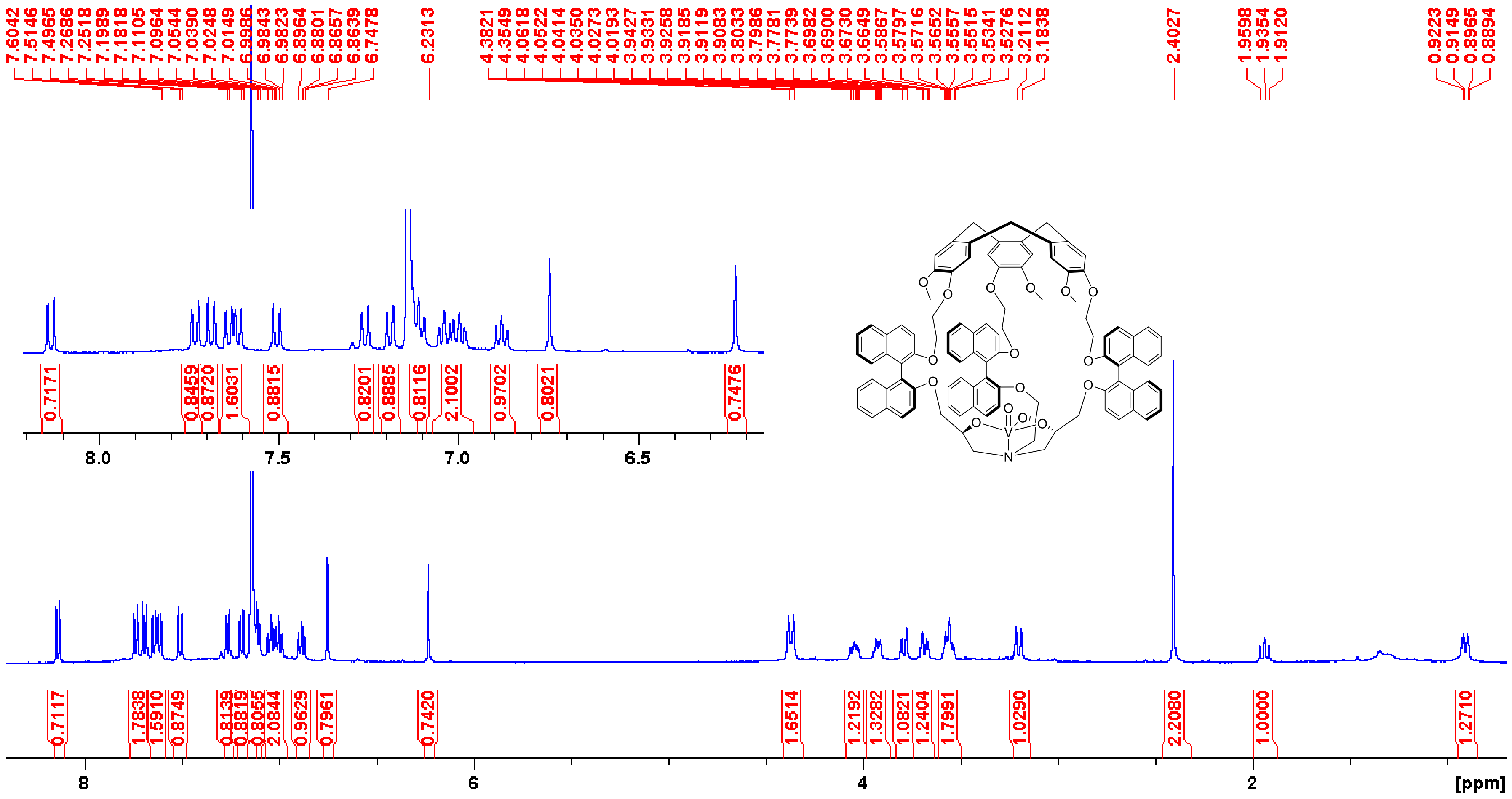

^{1}H NMR spectrum (C_6D_6, 500.1 MHz, 298K) of *M*-(*S*,*S*,*S*)-(*S*,*S*,*S*)-**2**.

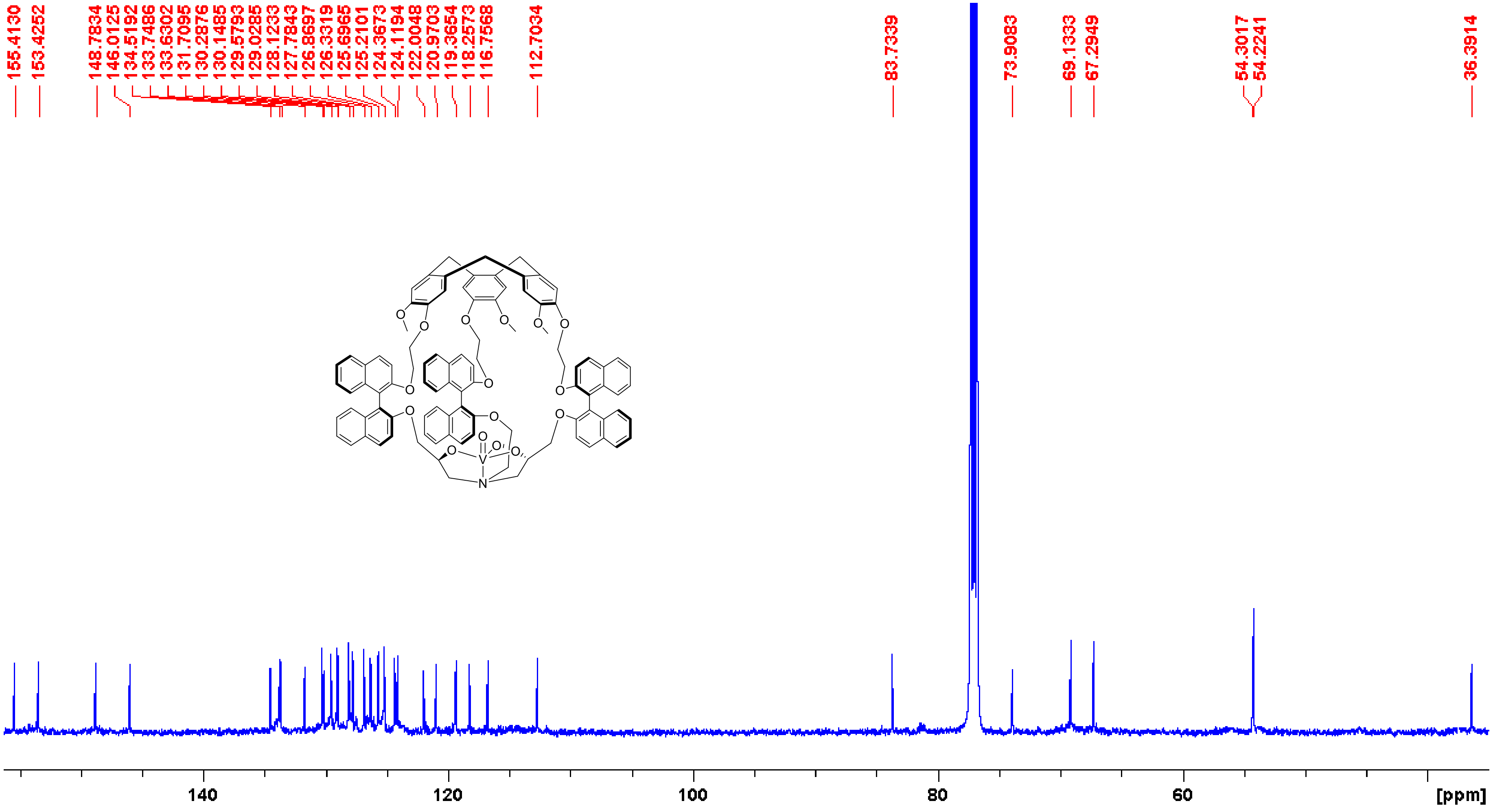

^{13}C NMR spectrum ($CDCl_3$, 125.8 MHz, 298K) of *M*-(*S*,*S*,*S*)-(*S*,*S*,*S*)-**2**.

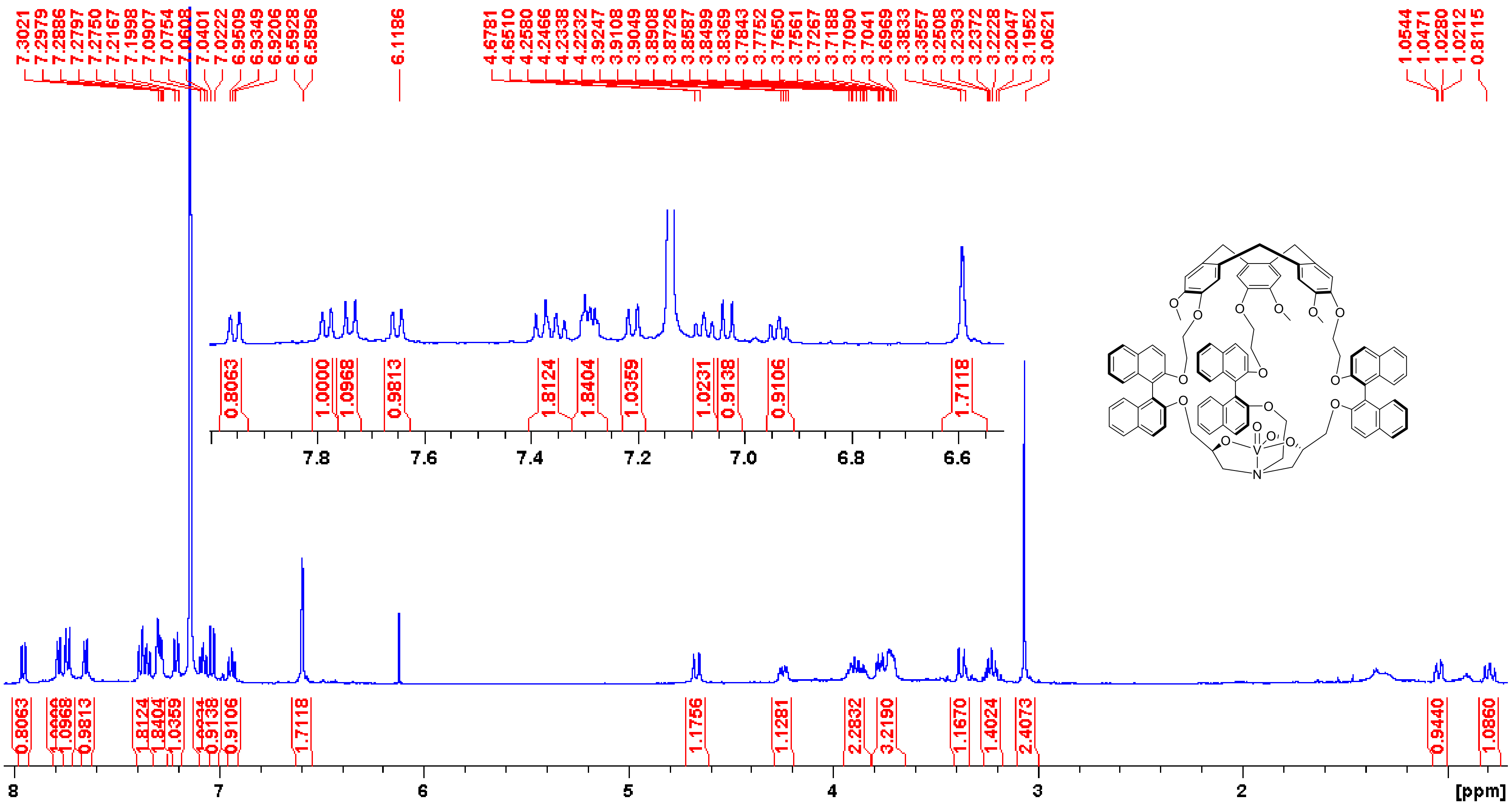

^{1}H NMR spectrum (C_6D_6, 500.1 MHz, 298K) of *P*-(*S*,*S*,*S*)-(*R*,*R*,*R*)-**2**.

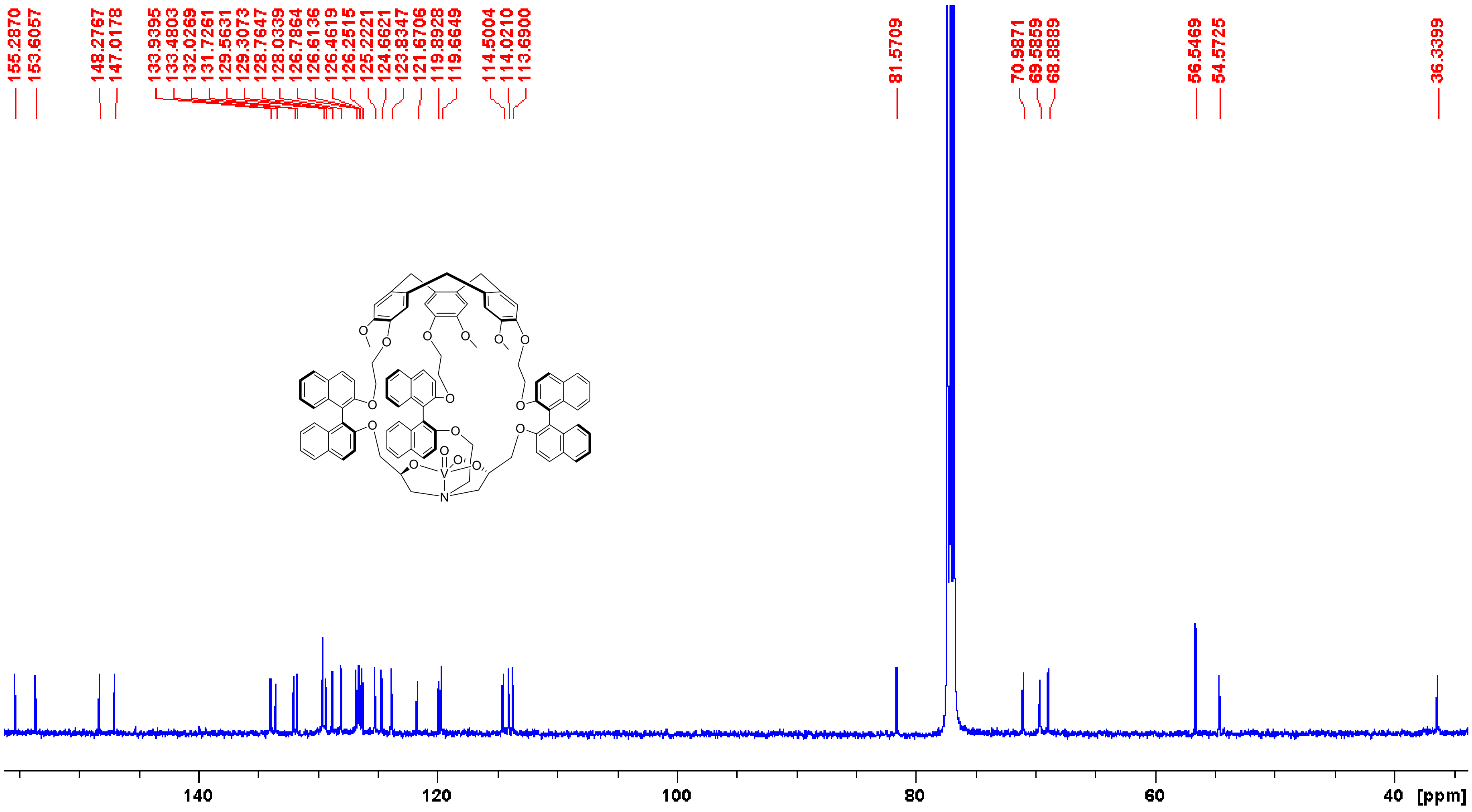

^{13}C NMR spectrum ($CDCl_3$, 125.8 MHz, 298K) of *P*-(*S*,*S*,*S*)-(*R*,*R*,*R*)-**2**.

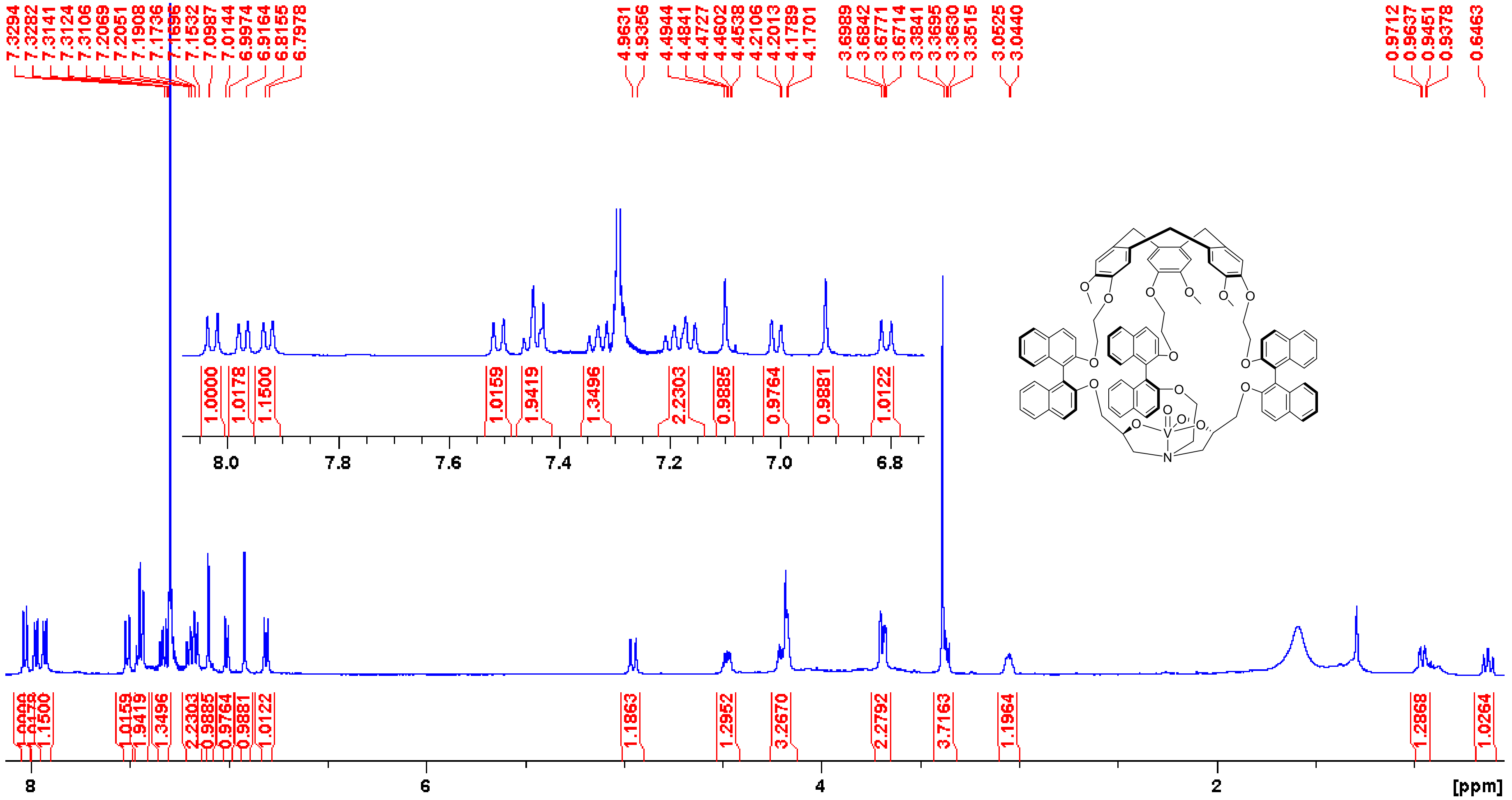

^{1}H NMR spectrum ($CDCl_3$, 500.1 MHz, 298K) of *M*-(*S*,*S*,*S*)-(*R*,*R*,*R*)-**2**.

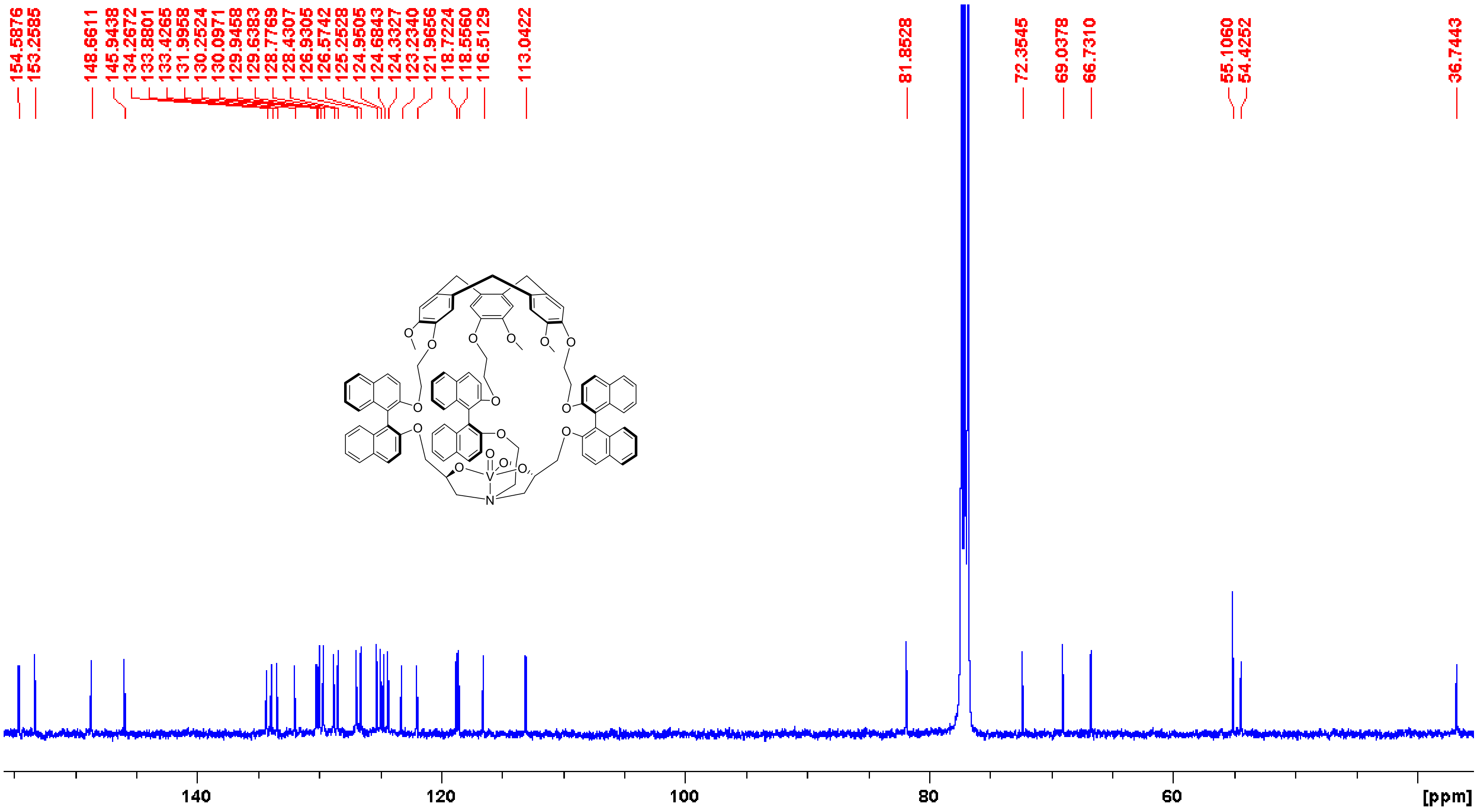

^{13}C NMR spectrum ($CDCl_3$, 125.8 MHz, 298K) of *M*-(*S*,*S*,*S*)-(*R*,*R*,*R*)-**2**.

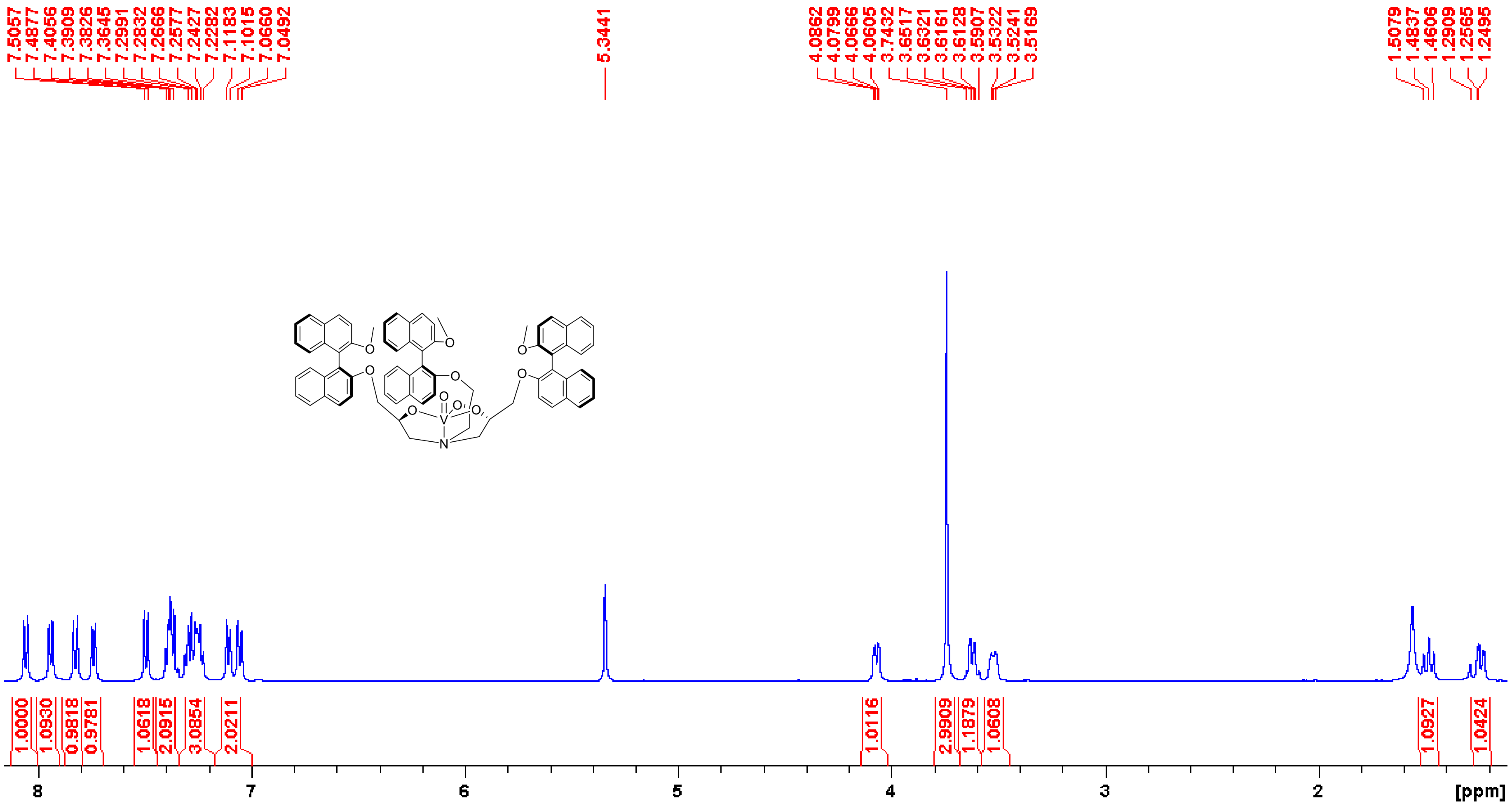

^{1}H NMR spectrum (CD_2Cl_2, 500.1 MHz, 298K) of (*S*,*S*,*S*)-(*S*,*S*,*S*)-**4**.

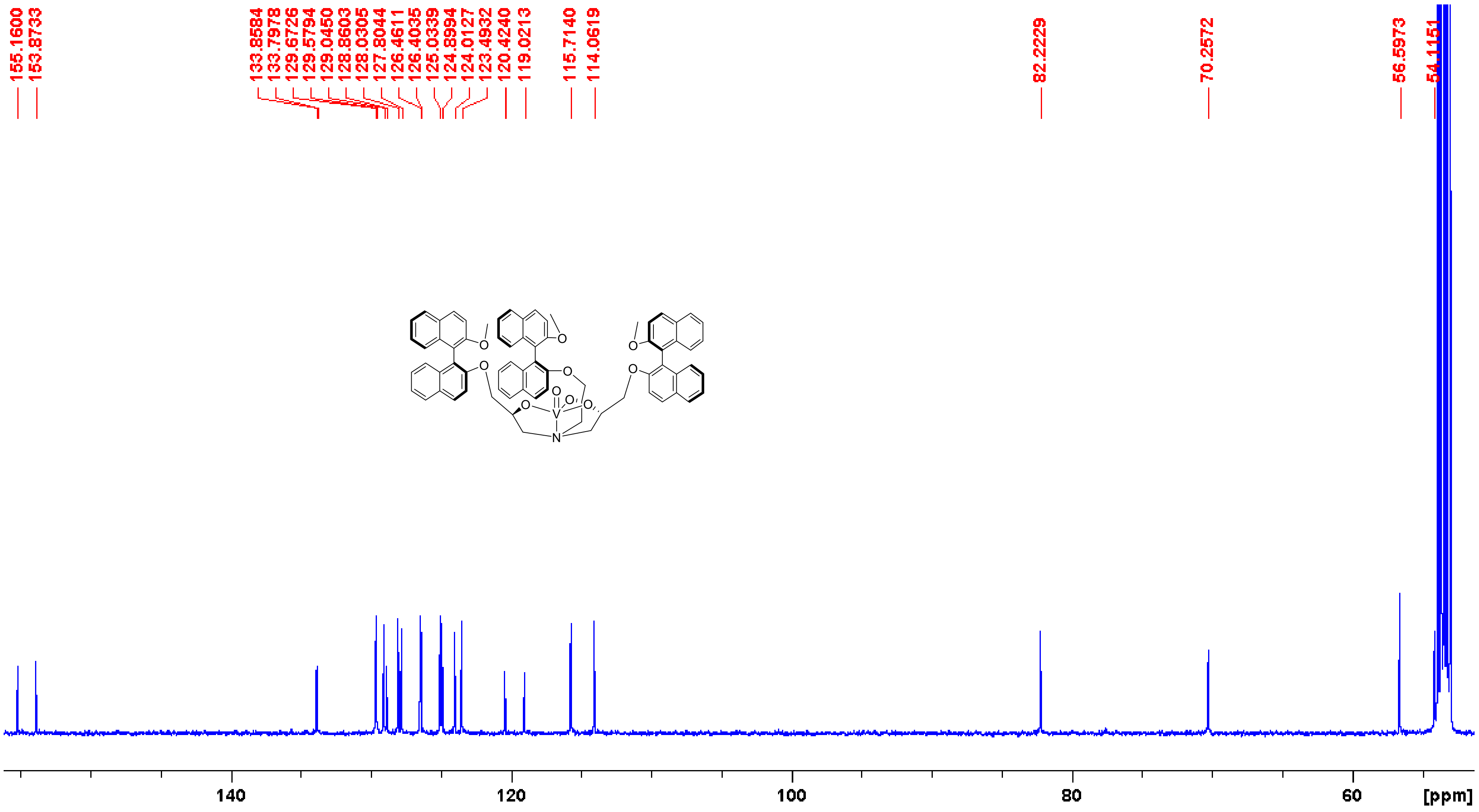

^{13}C NMR spectrum (CD_2Cl_2, 125.8 MHz, 298K) of (*S,S,S*)-(*S,S,S*)-**4**.

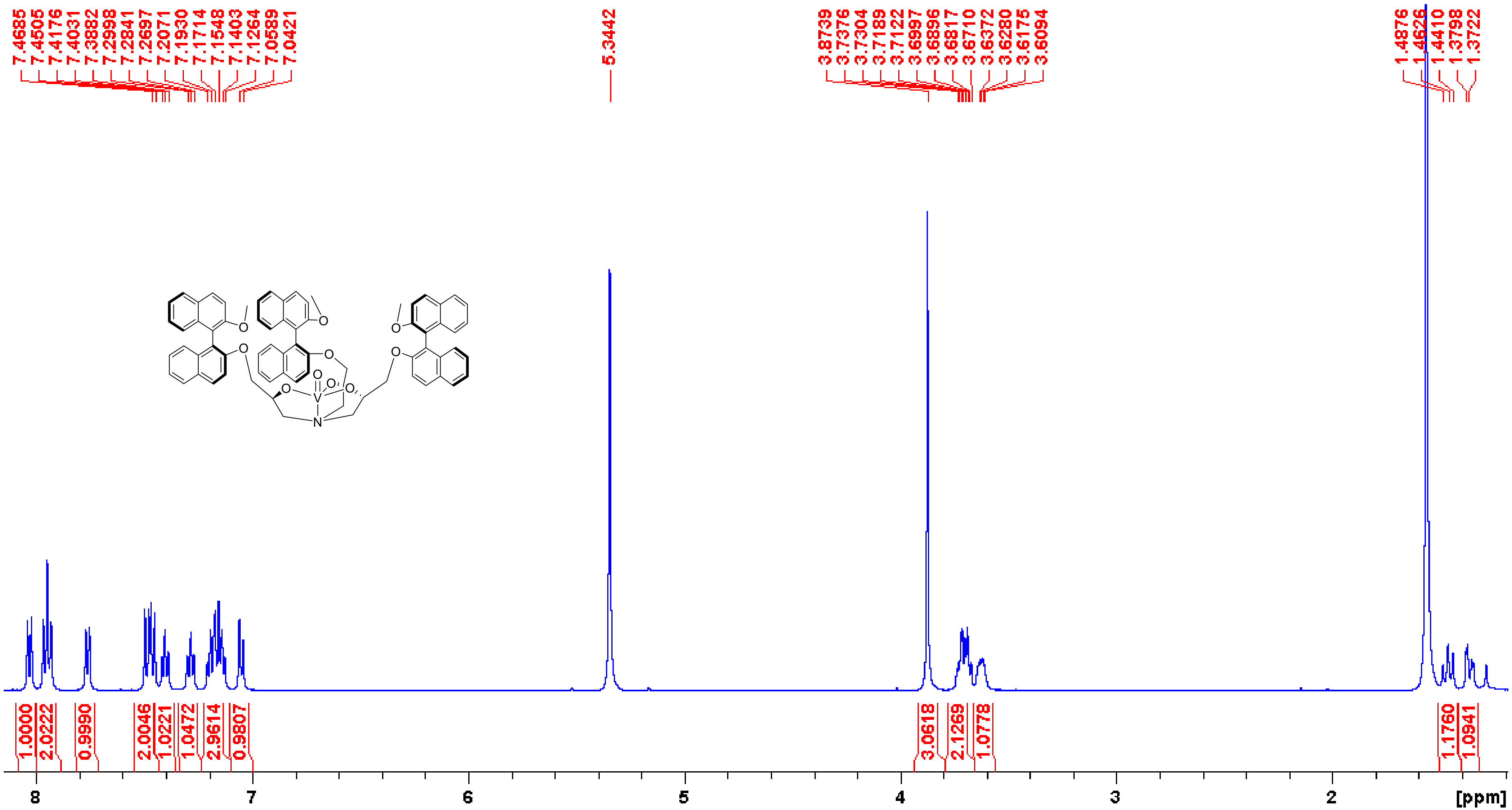

^{1}H NMR spectrum (CD_2Cl_2, 500.1 MHz, 298K) of (*S*,*S*,*S*)-(*R*,*R*,*R*)-**4**.

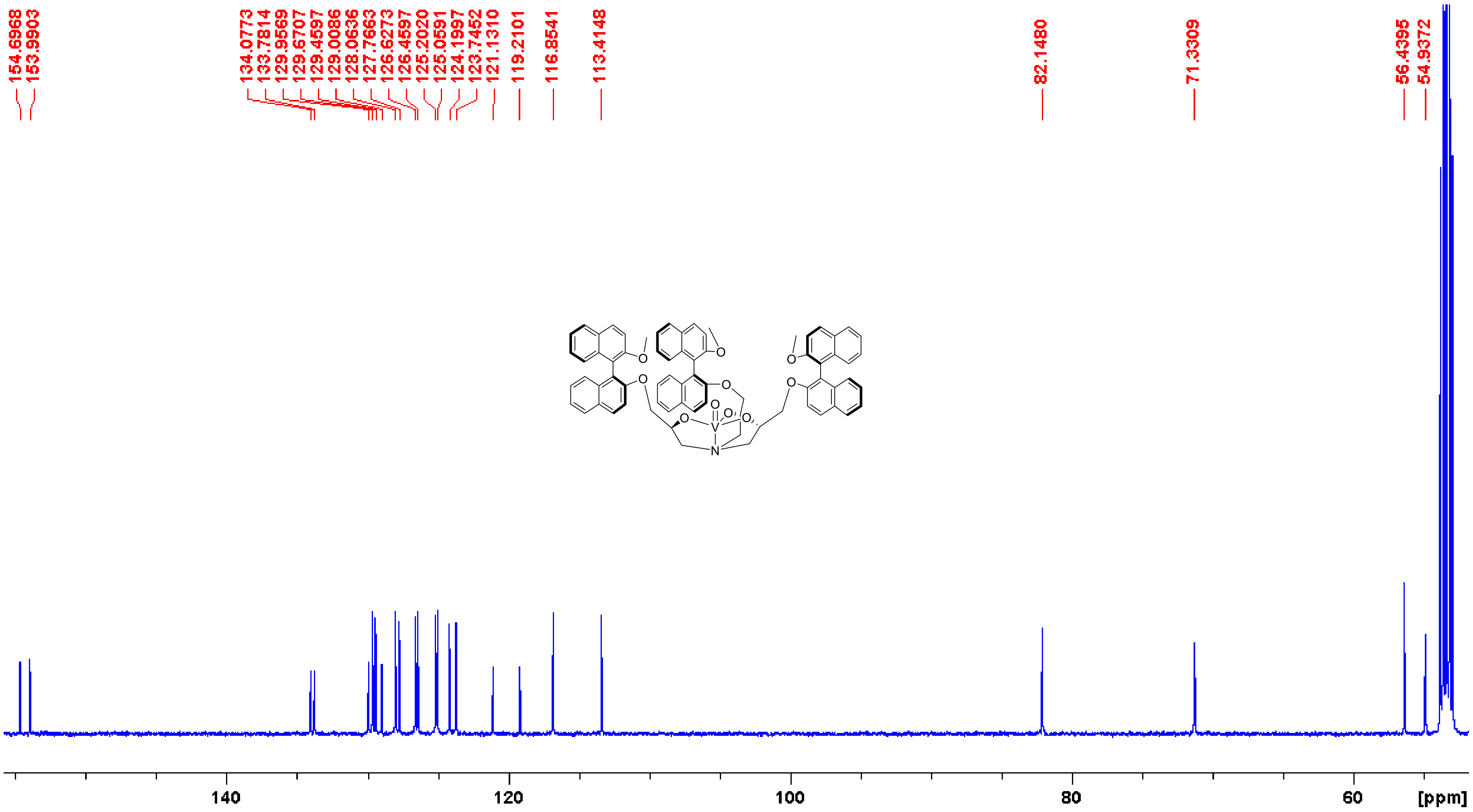

^{13}C NMR spectrum (CD_2Cl_2, 125.8 MHz, 298K) of (*S*,*S*,*S*)-(*R*,*R*,*R*)-**4**.

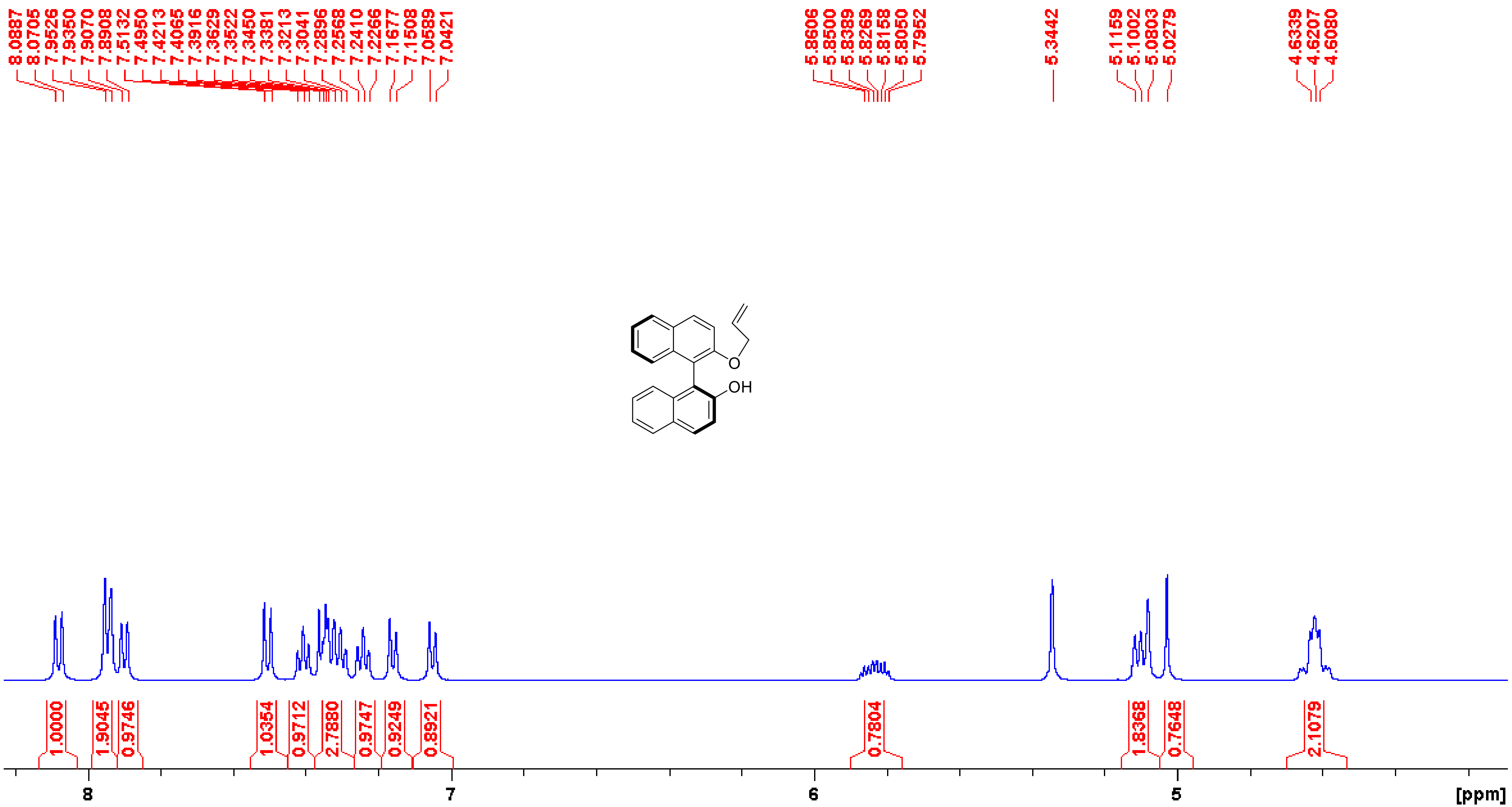

^{1}H NMR spectrum (CD_2Cl_2, 500.1 MHz, 298K) of *S*-**7**.

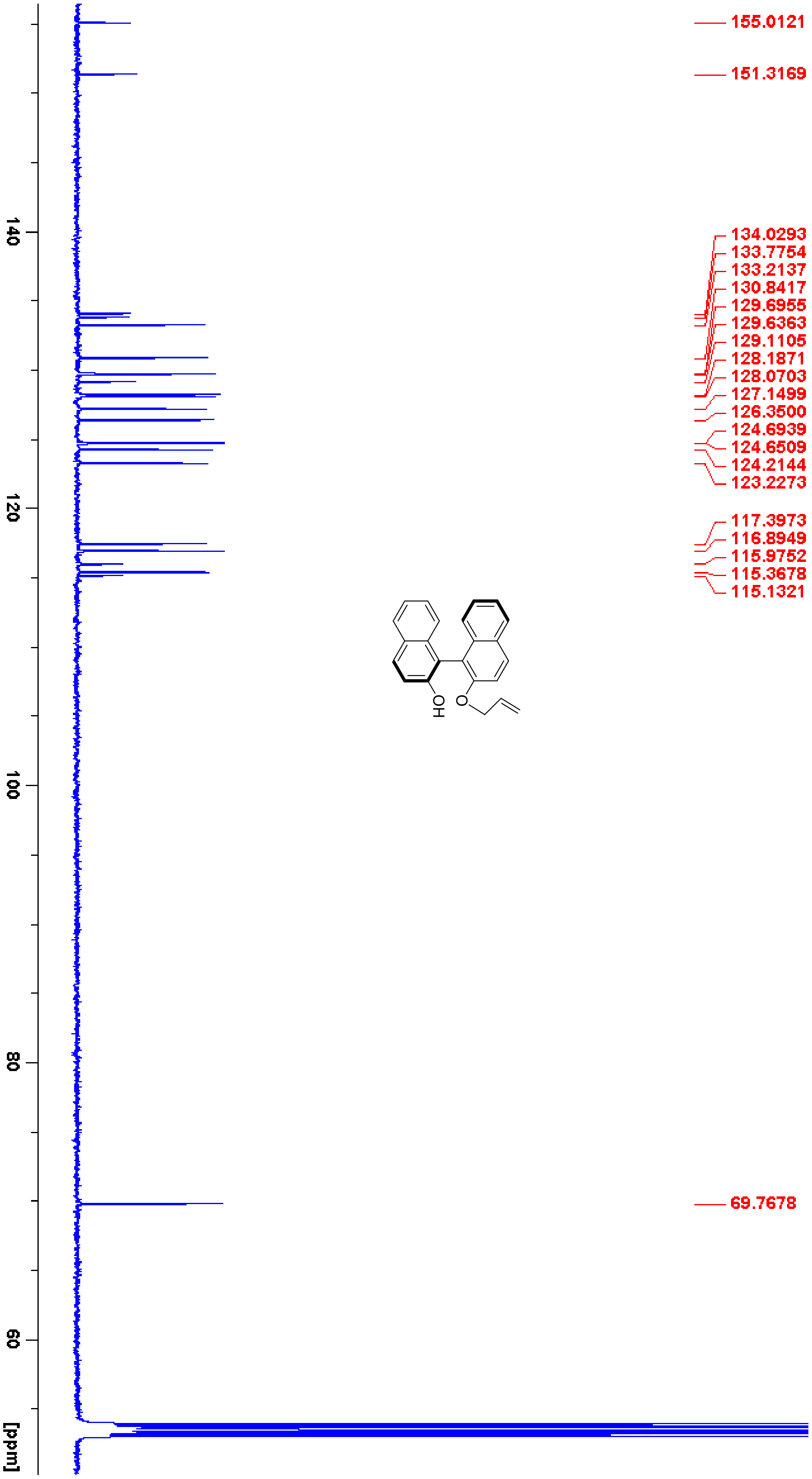

^{13}C NMR spectrum (CD_2Cl_2, 125.8 MHz, 298K) of *S*-7.

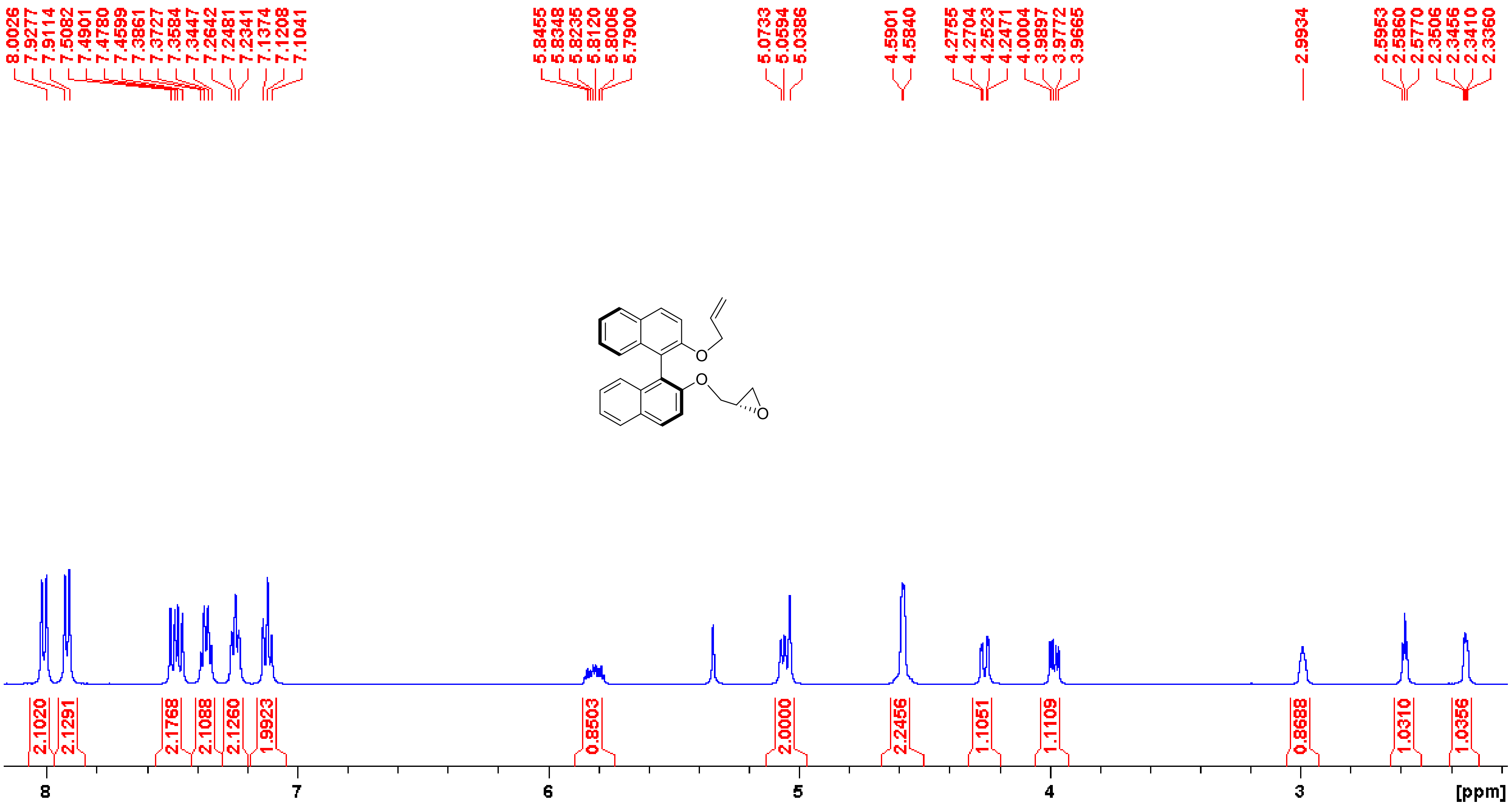

^{1}H NMR spectrum (CD_2Cl_2, 500.1 MHz, 298K) of *S*-*S*-**8**.

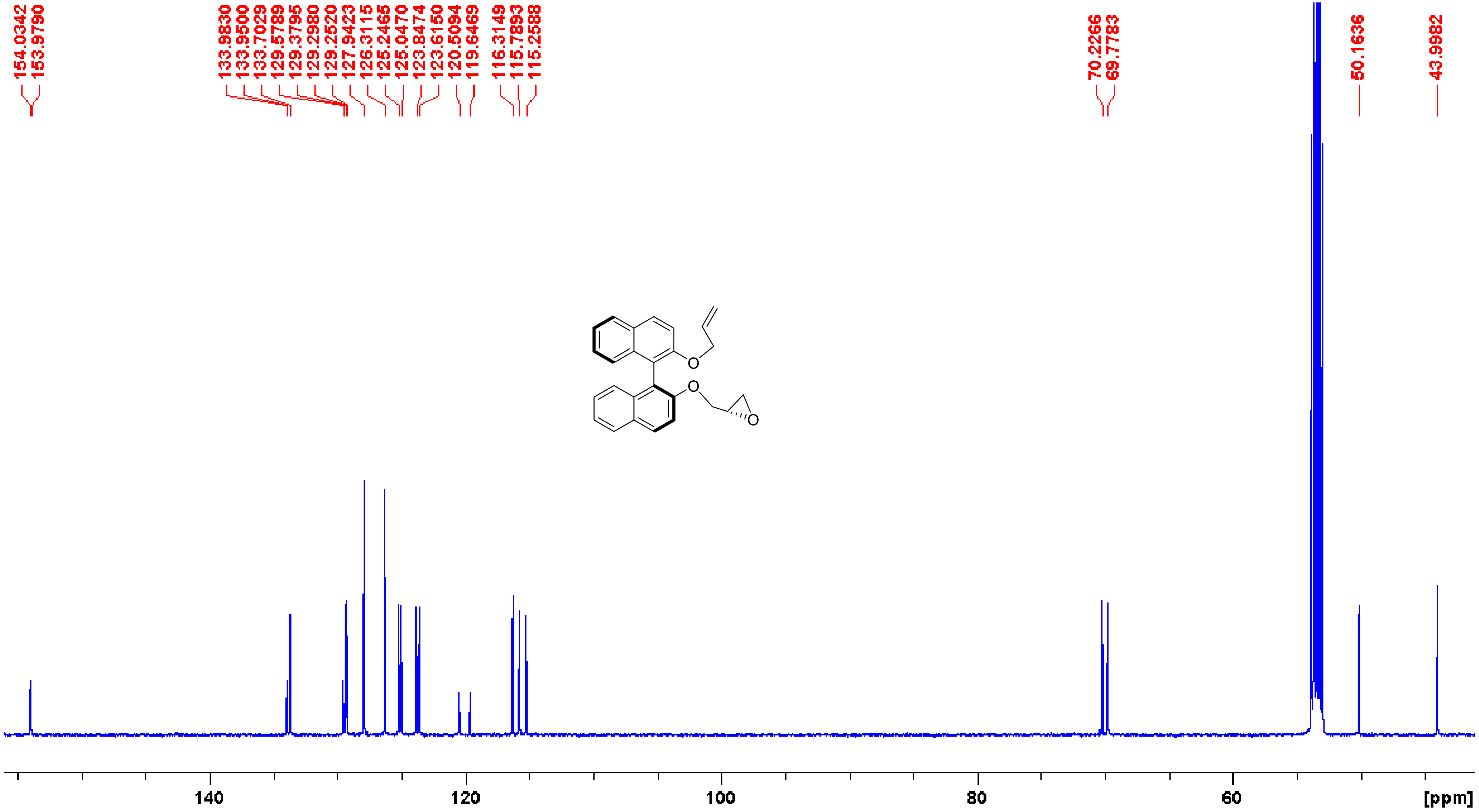

^{13}C NMR spectrum (CD_2Cl_2, 125.8 MHz, 298K) of *S*-*S*-**8**.

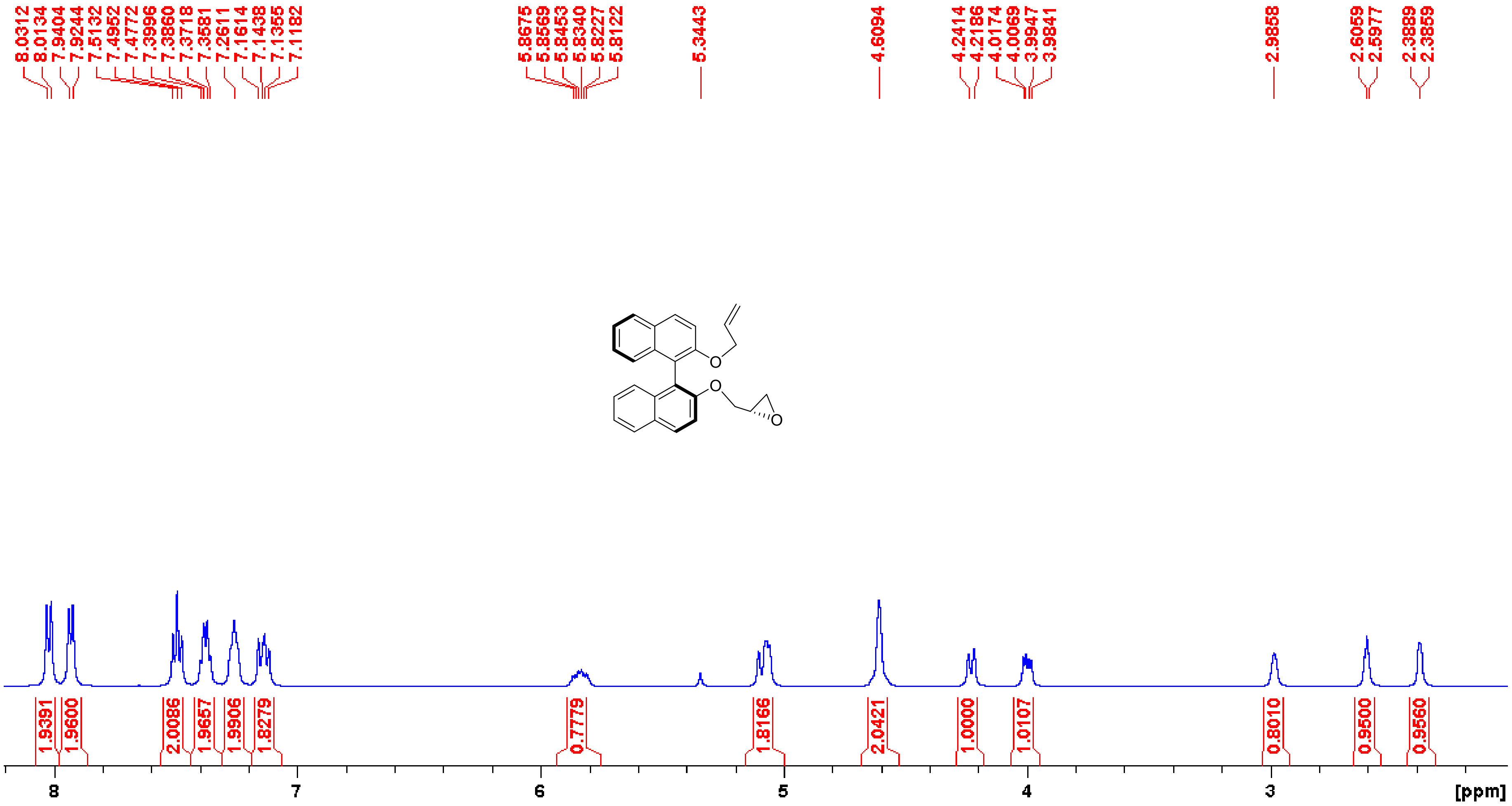

^{1}H NMR spectrum (CD_2Cl_2, 500.1 MHz, 298K) of *S*-*R*-**8**.

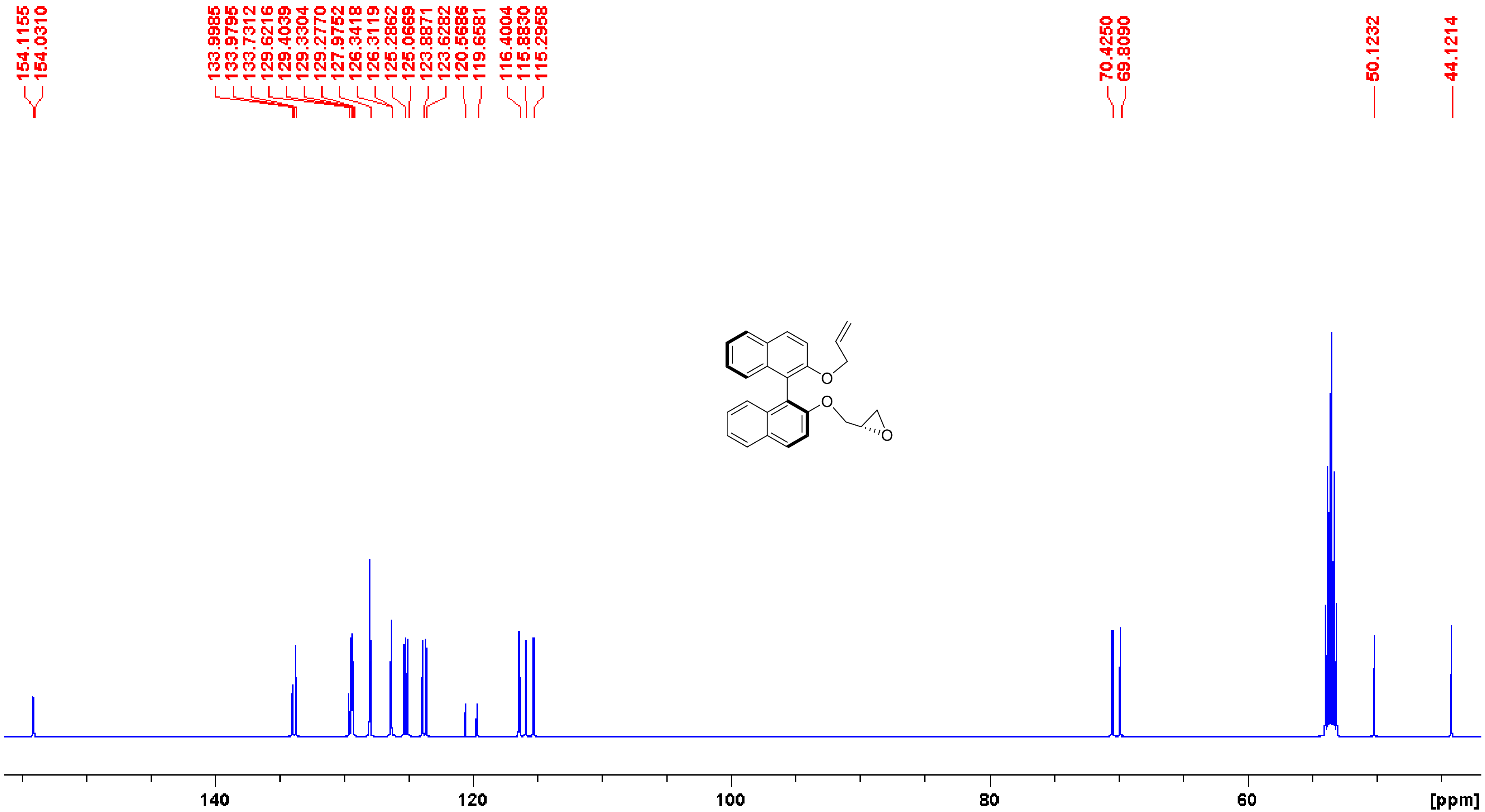

^{13}C NMR spectrum (CD_2Cl_2, 125.8 MHz, 298K) of *S*-*R*-**8**.

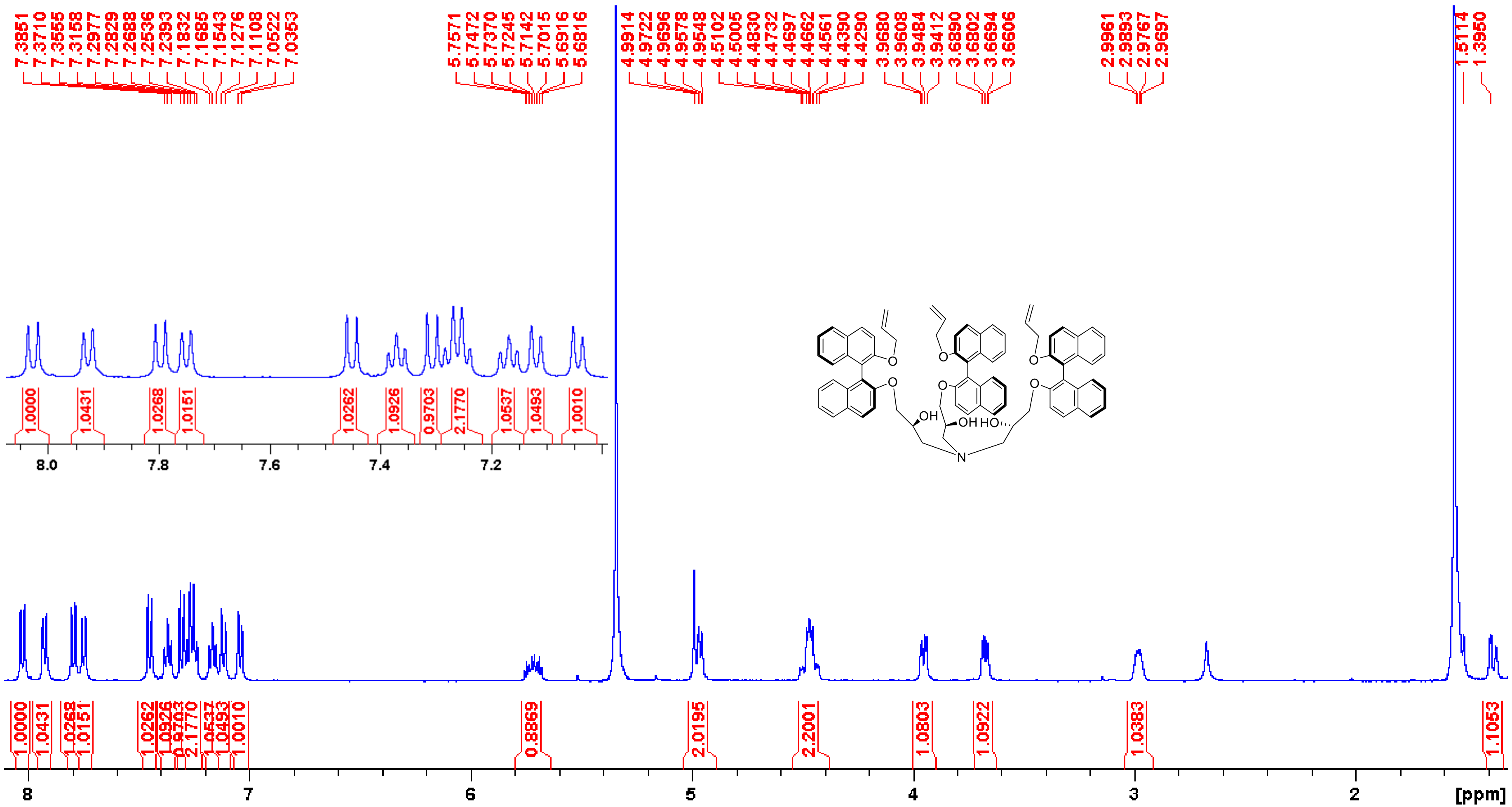

^{1}H NMR spectrum (CD_2Cl_2, 500.1 MHz, 298K) of (*S*,*S*,*S*)-(*S*,*S*,*S*)-**10**.

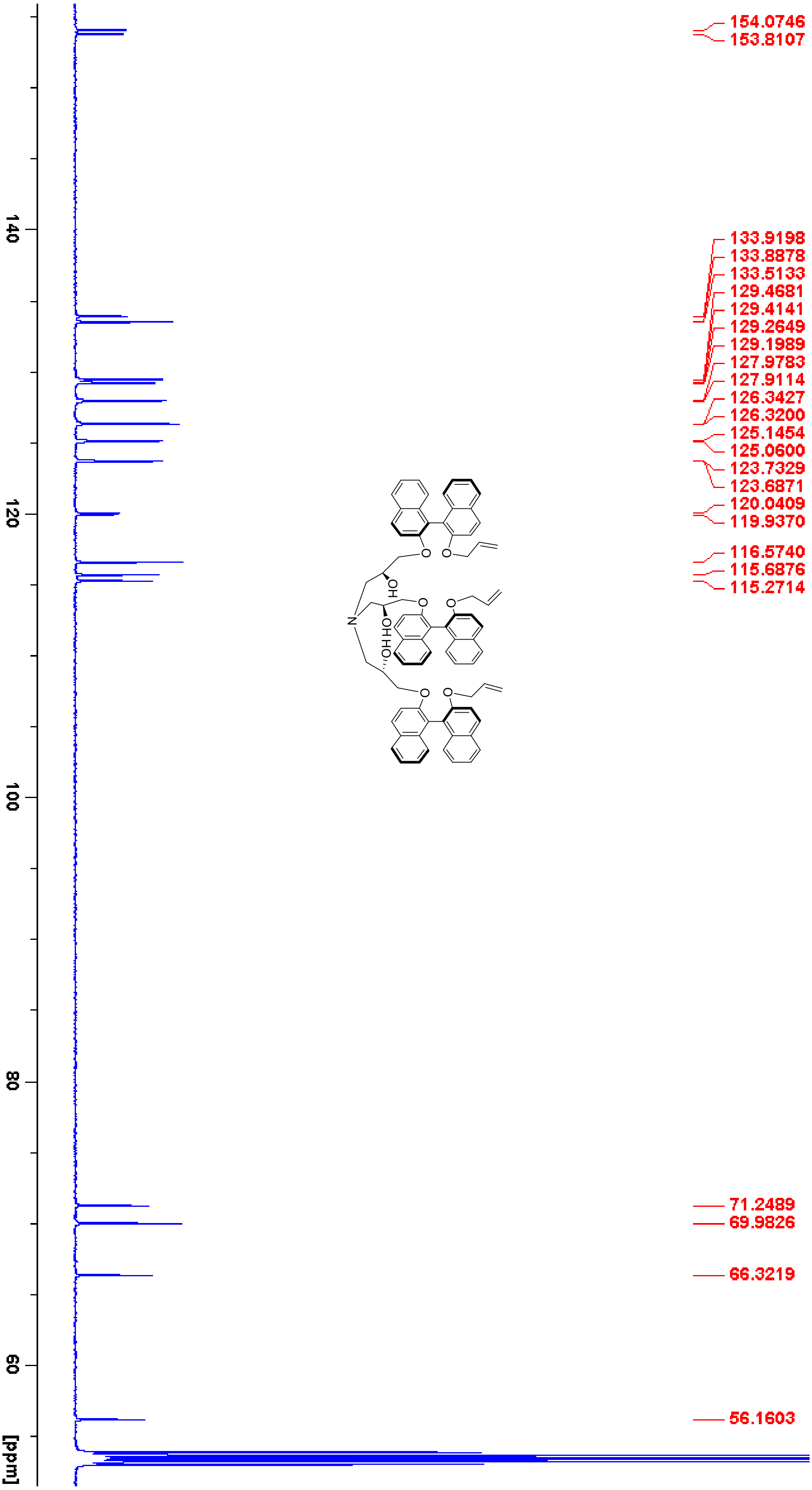

^{13}C NMR spectrum (CD_2Cl_2, 125.8 MHz, 298K) of (*S,S,S*)-(*S,S,S*)-**10**.

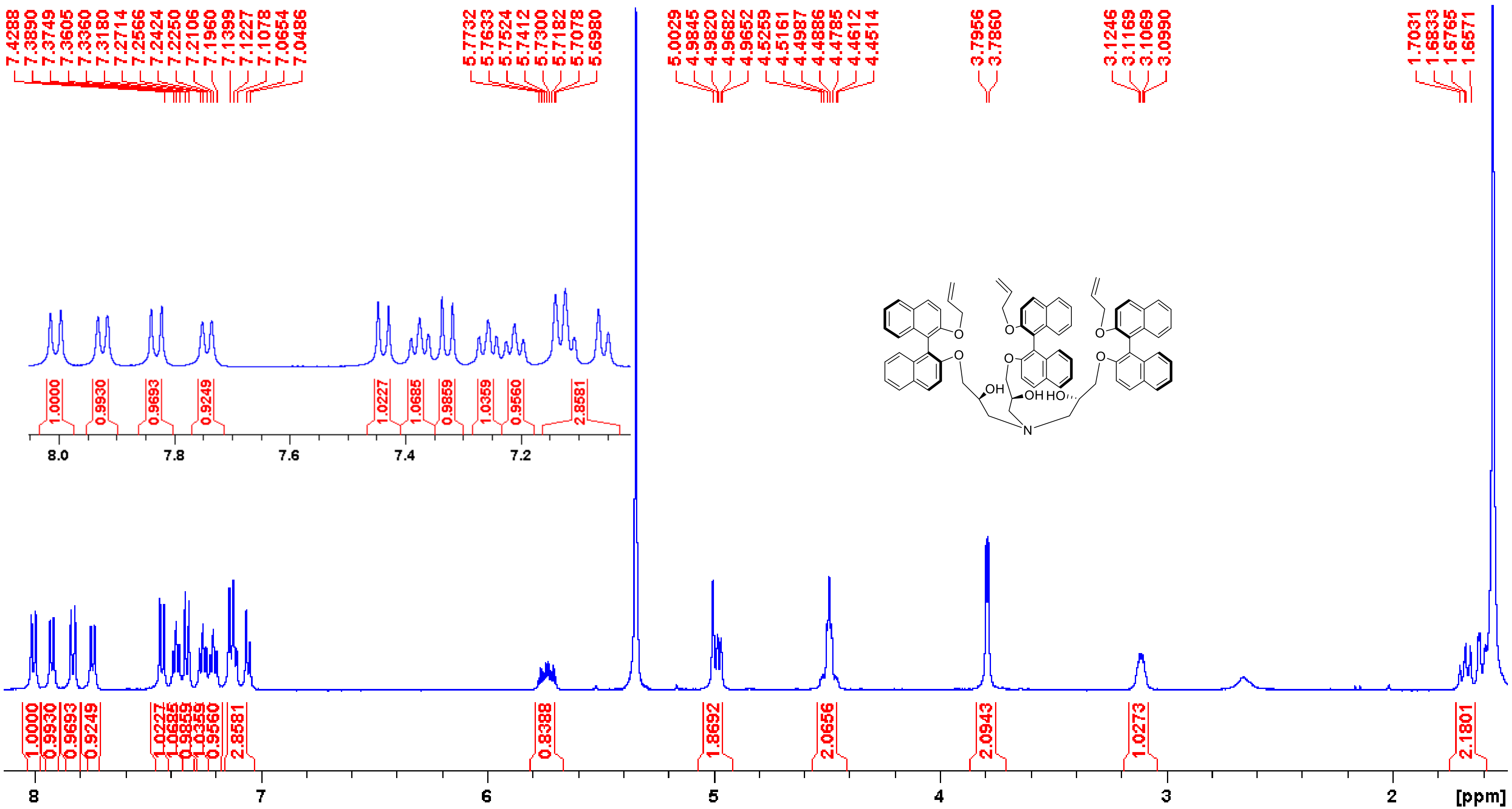

^{1}H NMR spectrum (CD_2Cl_2, 500.1 MHz, 298K) of (*S*,*S*,*S*)-(*R*,*R*,*R*)-**10**.

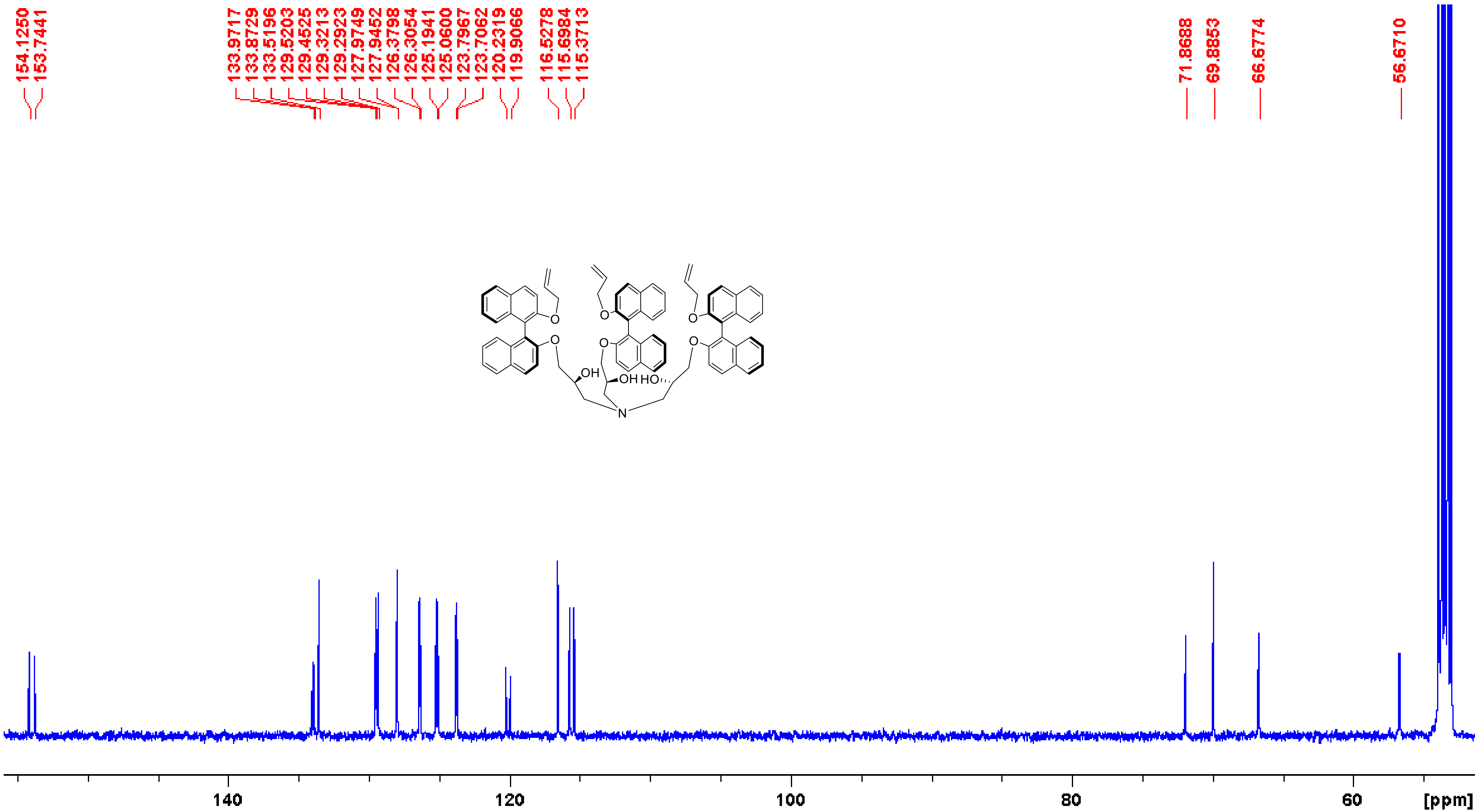

^{13}C NMR spectrum (CD_2Cl_2, 125.8 MHz, 298K) of (*S*,*S*,*S*)-(*R*,*R*,*R*)-**10**.

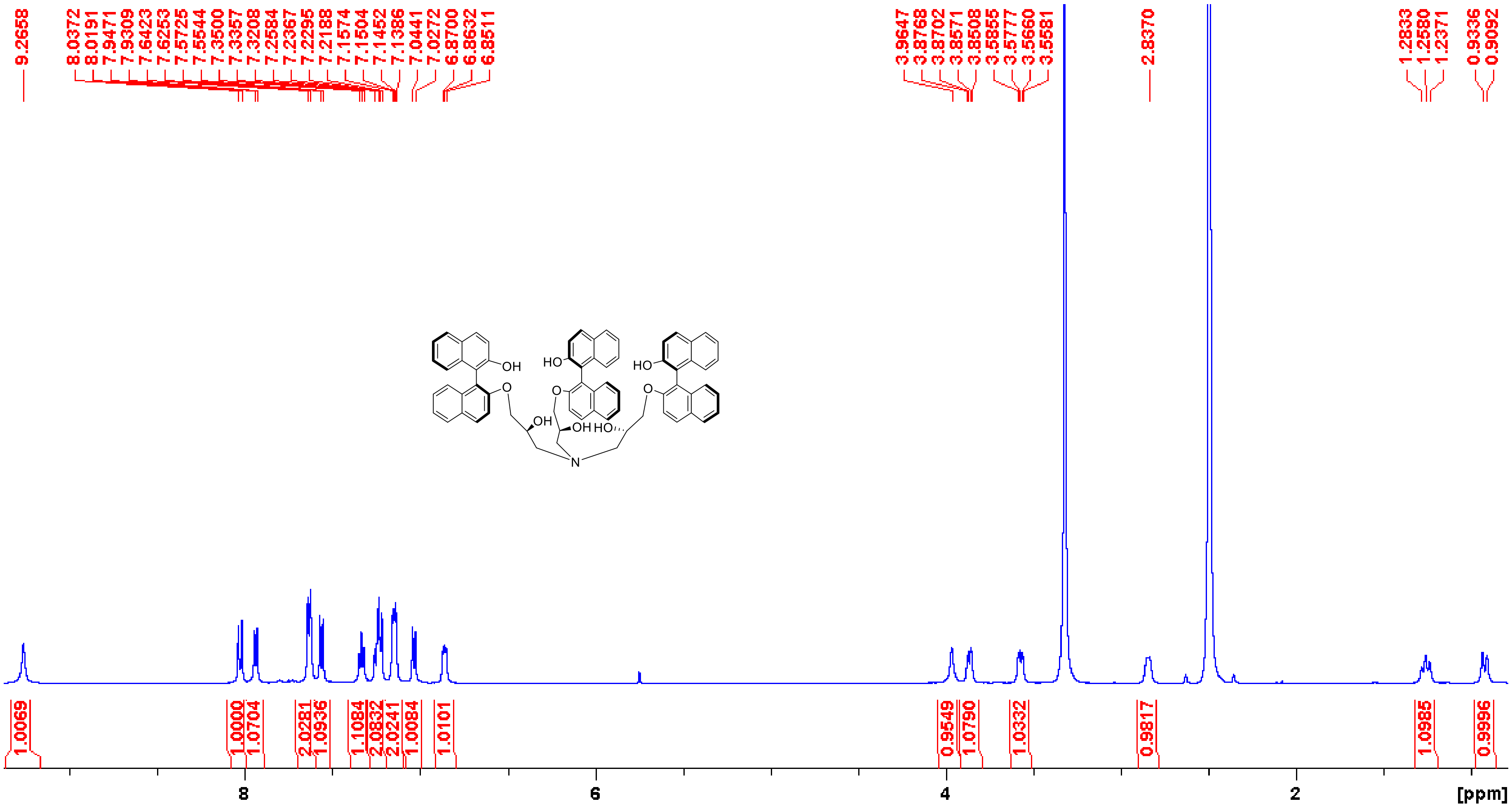

^{1}H NMR spectrum (DMSO-d_6, 500.1 MHz, 298K) of (*S*,*S*,*S*)-(*S*,*S*,*S*)-**11**.

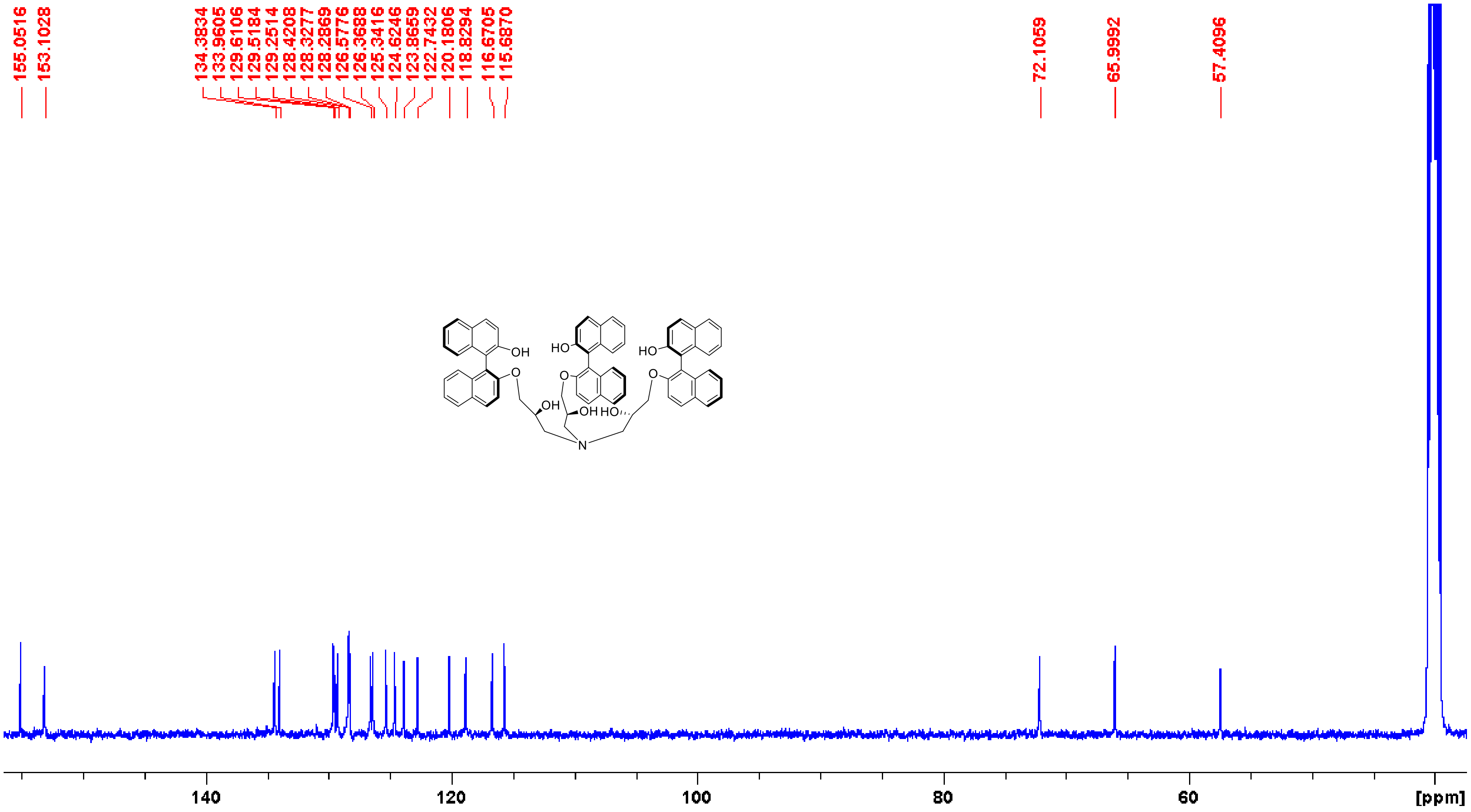

^{13}C NMR spectrum (DMSO-d_6, 125.8 MHz, 298K) of (*S*,*S*,*S*)-(*S*,*S*,*S*)-**11**.

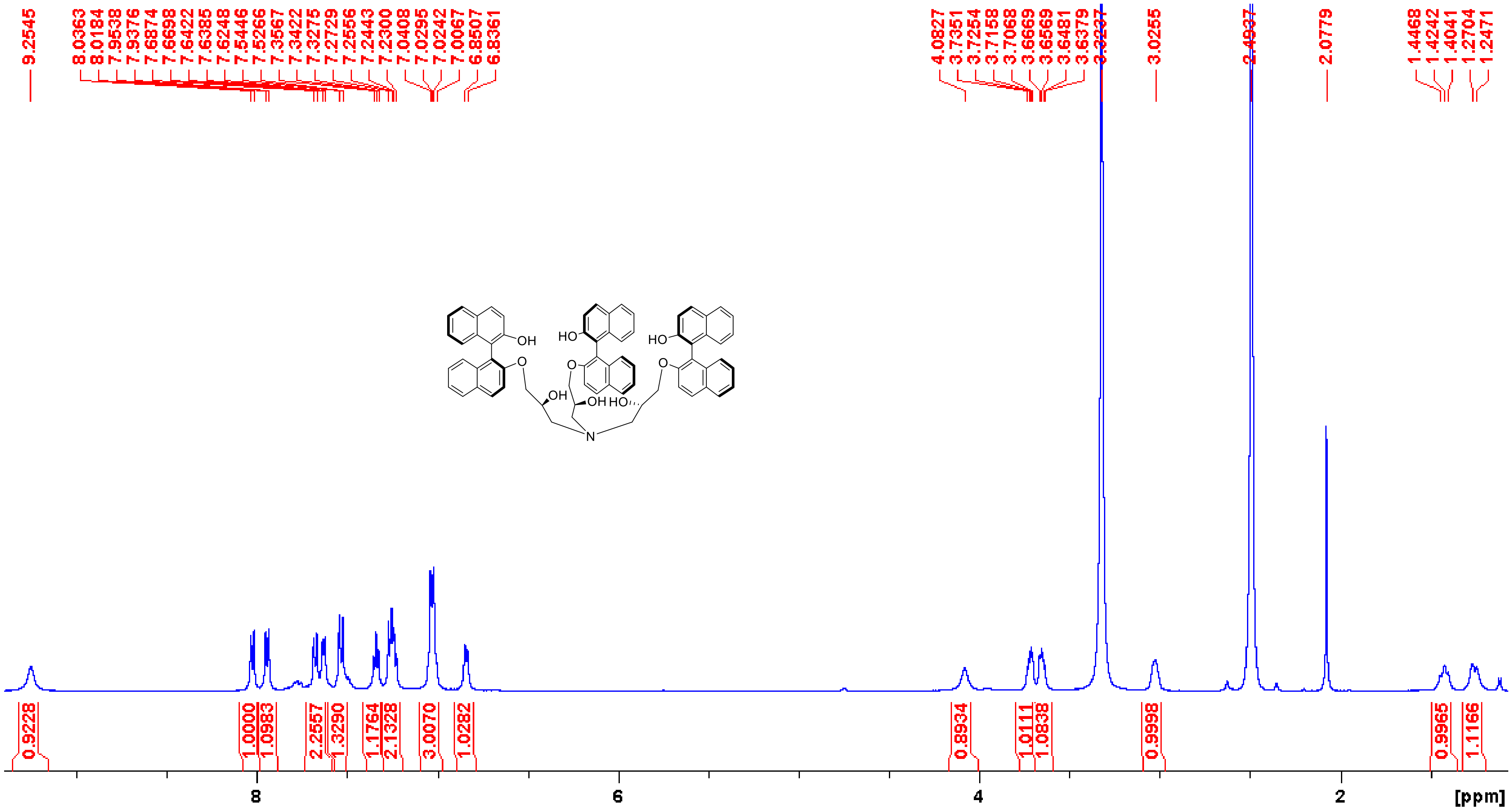

^{1}H NMR spectrum (DMSO-d_6, 500.1 MHz, 298K) of (*S*,*S*,*S*)-(*R*,*R*,*R*)-**11**.

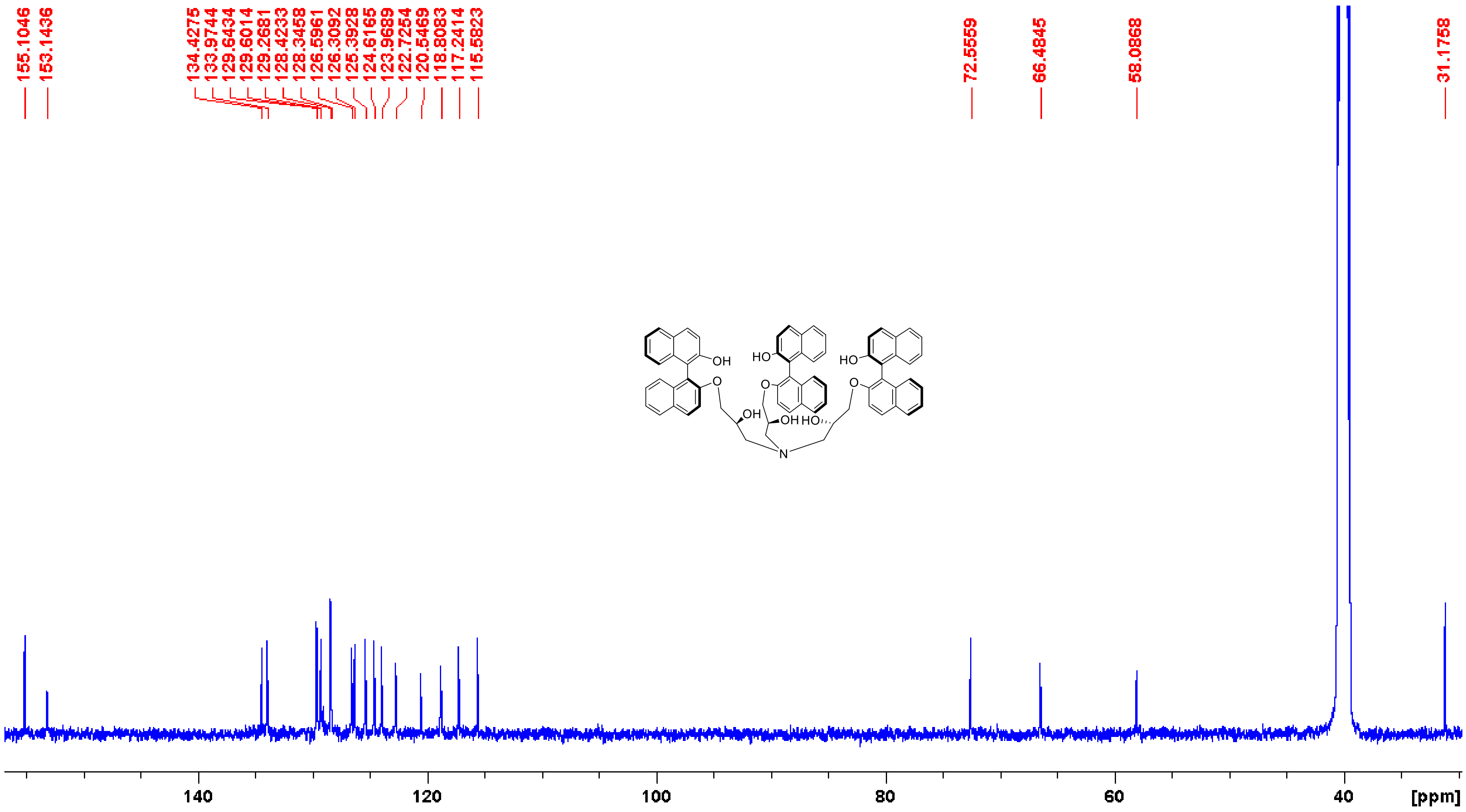

^{13}C NMR spectrum (DMSO-d_6, 125.8 MHz, 298K) of (*S*,*S*,*S*)-(*R*,*R*,*R*)-**11**.

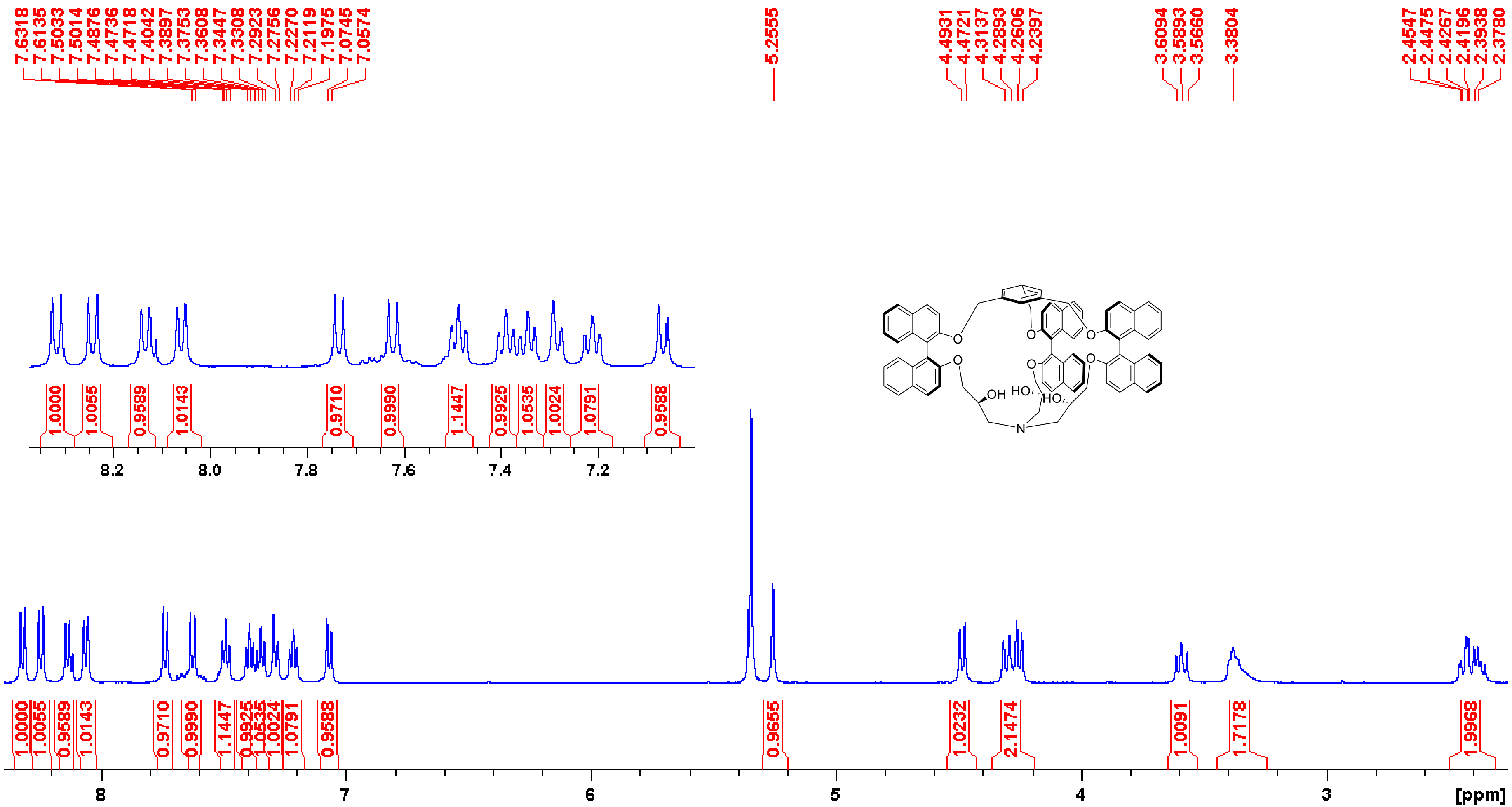

^{1}H NMR spectrum (CD_2Cl_2, 500.1 MHz, 298K) of (*S*,*S*,*S*)-(*S*,*S*,*S*)-**12**.

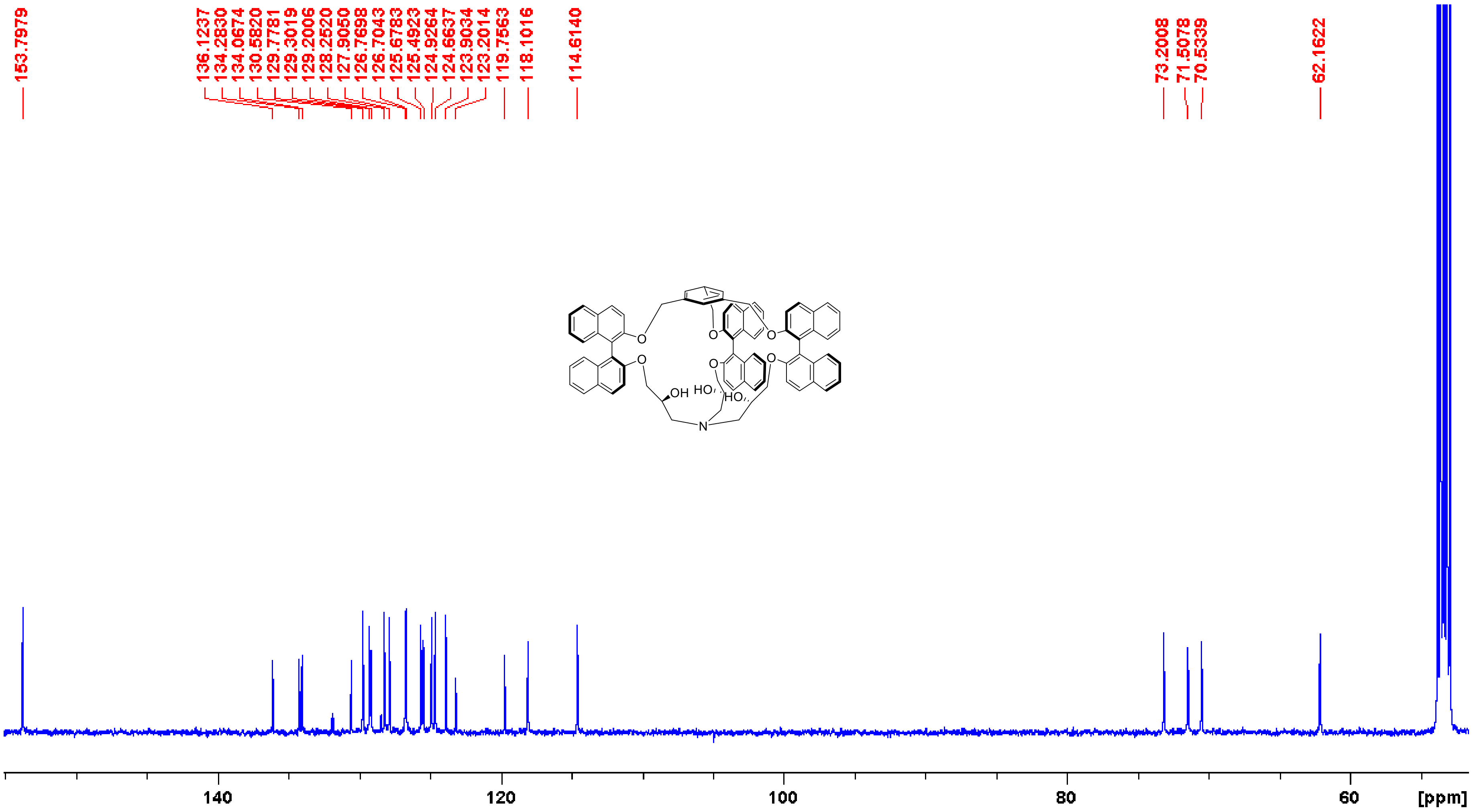

^{13}C NMR spectrum (CD_2Cl_2, 125.8 MHz, 298K) of (*S*,*S*,*S*)-(*S*,*S*,*S*)-**12**.

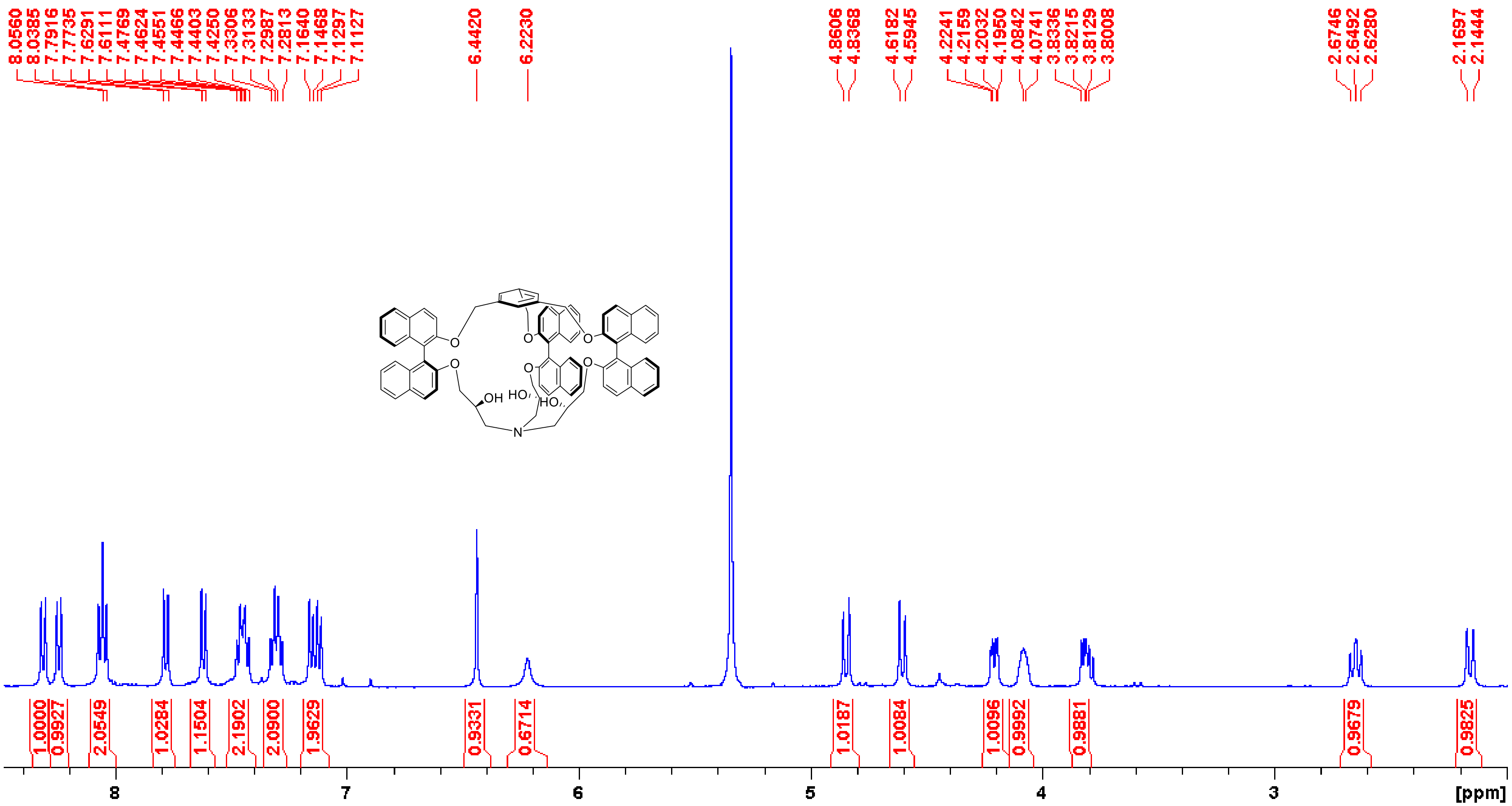

^{1}H NMR spectrum (CD_2Cl_2, 500.1 MHz, 298K) of (*S*,*S*,*S*)-(*R*,*R*,*R*)-**12**.

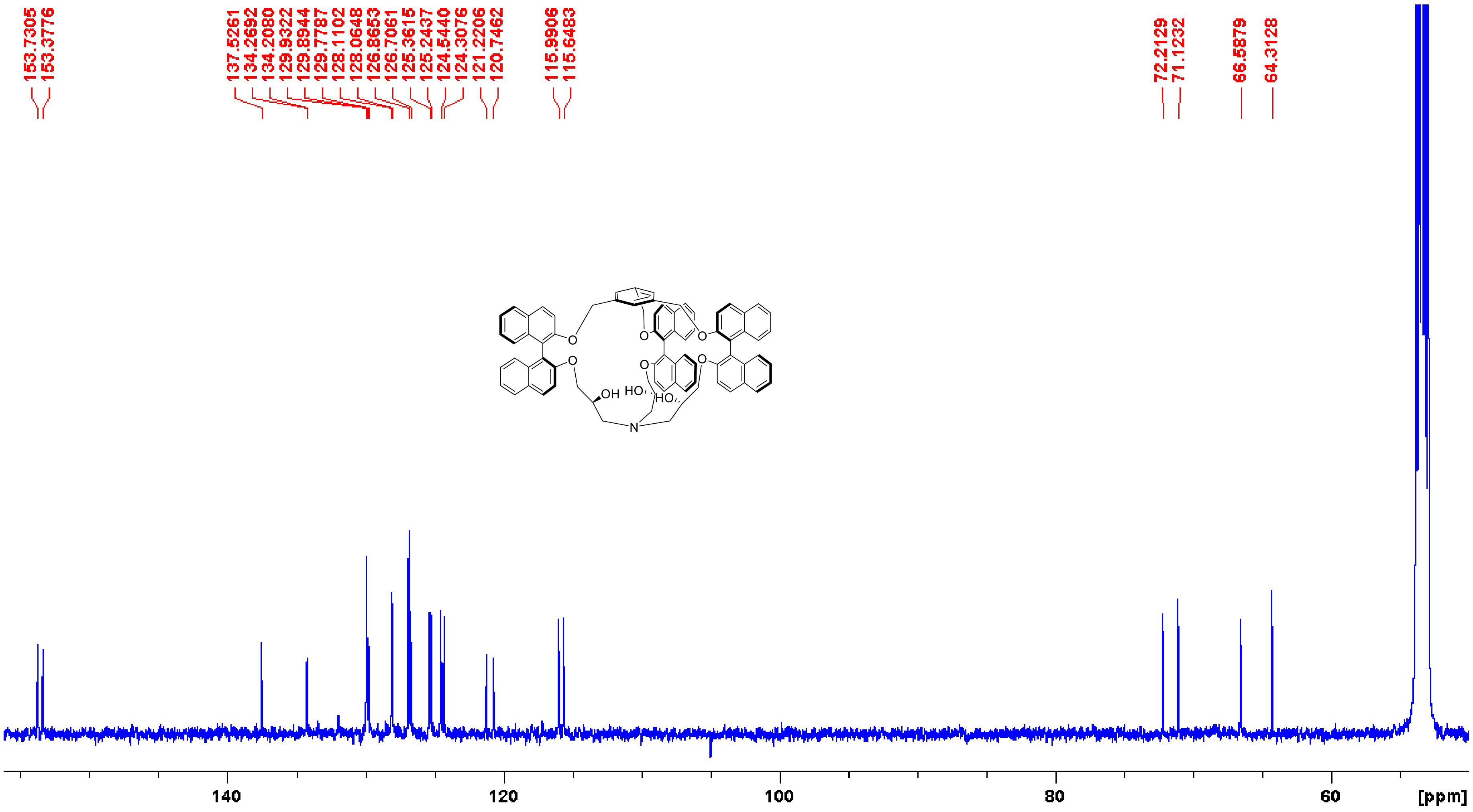

^{13}C NMR spectrum (CD_2Cl_2, 125.8 MHz, 298K) of (*S*,*S*,*S*)-(*R*,*R*,*R*)-**12**.
